# Cysteine peptidases and their inhibitors in *Tetranychus urticae*: a comparative genomic approach

**DOI:** 10.1186/1471-2164-13-307

**Published:** 2012-07-11

**Authors:** María Estrella Santamaría, Pedro Hernández-Crespo, Félix Ortego, Vojislava Grbic, Miodrag Grbic, Isabel Diaz, Manuel Martinez

**Affiliations:** 1 Department of Biology WSC 339/341, The University of Western Ontario, 1151 Richmond St, London, ON N6A 5B7, Canada; 2 Laboratorio de Interacción Planta-Insecto, Departamento de Biología Medioambiental, Centro de Investigaciones Biológicas, CSIC, Ramiro de Maeztu, 9, 28040, Madrid, Spain; 3 Centro de Biotecnología y Genómica de Plantas, Universidad Politécnica de Madrid, Campus Montegancedo, 28223-Pozuelo de Alarcón, Madrid, Spain

**Keywords:** Comparative genomics, Cystatins, Cysteine peptidases, Feeding, *Tetranychus urticae*, Thyropins

## Abstract

**Background:**

Cysteine peptidases in the two-spotted spider mite *Tetranychus urticae* are involved in essential physiological processes, including proteolytic digestion. Cystatins and thyropins are inhibitors of cysteine peptidases that modulate their activity, although their function in this species has yet to be investigated. Comparative genomic analyses are powerful tools to obtain advanced knowledge into the presence and evolution of both, peptidases and their inhibitors, and could aid to elucidate issues concerning the function of these proteins.

**Results:**

We have performed a genomic comparative analysis of cysteine peptidases and their inhibitors in *T. urticae* and representative species of different arthropod taxonomic groups. The results indicate: i) clade-specific proliferations are common to C1A papain-like peptidases and for the I25B cystatin family of inhibitors, whereas the C1A inhibitors thyropins are evolutionarily more conserved among arthropod clades; ii) an unprecedented extensive expansion for C13 legumain-like peptidases is found in *T. urticae*; iii) a sequence-structure analysis of the spider mite cystatins suggests that diversification may be related to an expansion of their inhibitory range; and iv) an *in silico* transcriptomic analysis shows that most cathepsin B and L cysteine peptidases, legumains and several members of the cystatin family are expressed at a higher rate in *T. urticae* feeding stages than in embryos.

**Conclusion:**

Comparative genomics has provided valuable insights on the spider mite cysteine peptidases and their inhibitors. Mite-specific proliferations of C1A and C13 peptidase and I25 cystatin families and their over-expression in feeding stages of mites fit with a putative role in mite’s feeding and could have a key role in its broad host feeding range.

## Background

The two-spotted spider mite, *Tetranychus urticae*, is one of the main pests of agricultural crops due to its broad host range. This polyphagous species feeds on more than 1,100 plant species, from which about 150 are of great economic value. Thus, it represents a very important pest for field and greenhouse crops, ornamentals, annual and perennial plants all over the world. *T. urticae* has one of the smallest known animal genomes of about 90Mbp with over 18,000 genes identified. Genome features, such as large expansion of gene families associated with digestion and detoxification of plant secondary compounds are consistent with the spider mite’s wide host feeding range [1].

Regarding mite digestive physiology, mites use both extracellular and intracellular digestion, with the latter occurring in gut wall-derived epithelial cells that ingest and digest food particles that can be free floating [2,3]. The midgut is the site for synthesis and secretion of digestive enzymes and absorption of nutrients. Processed food and epithelial cells pass into the posterior midgut, are subsequently compacted in the hindgut and excreted as faecal pellets [3]. Mite species that feed on plants rely mostly on cysteine peptidase activities for the digestion of dietary proteins [4,5].

Cysteine peptidases are enzymes that hydrolyse peptide bonds using a catalytic cysteine. The MEROPS database contains all the existing peptidases grouped in clans [6]. Clans represent one or more families that show evidence of their evolutionary relationship by their similar tertiary structures, or when structures are not available, by the order of catalytic-site residues in the polypeptide chain and often by common sequence motifs around the catalytic residues. At present, among the 72 families of cysteine peptidases identified, 43 are included in 8 clans exclusively formed by cysteine peptidases, 13 are distributed in three clans that comprise peptidases with different catalytic mechanisms, and 16 are not enclosed in any determined clan. Most of the cysteine peptidases characterized in arthropods belong to the papain-like family (C1A), although members of the legumain (C13), calpain (C2), caspase (C14) or separase (C50) families have also been reported.

Arthropod papain-like cysteine peptidases are homologous to mammalian cathepsins, which are present in lysosomes, but can also be localized in extracellular spaces [7]. In mites and insect species belonging to the orders Coleoptera, Hemiptera and Homoptera most C1A peptidases, particularly cathepsins L and B-like, are involved in the digestion process [8-10]. Besides, they are implicated in other physiological processes in arthropods, such as embryogenesis or metamorphosis [11-14]. For *T. urticaein vitro* assays determined that major protease activity in extracts relies on papain-like cysteine type protease activity [4], which match up with the proliferation of this gene family in the spider mite genome [1]. In addition, a multigene family of legumain genes was also found in the spider mite genome [1], which could have a role in feeding similar to that observed for a legumain peptidase related to the digestive process of the hard tick *Ixodes ricinus*[15].

The most known inhibitors of C1A and C13 peptidases are the members of the I25 cystatin superfamily. In arthropods, the two ancestral eukaryotic lineages of the cystatin superfamily, stefins (I25A) and cystatins (I25B), have been reported [16]. Stefins are single copy intracellular inhibitors without disulphide bridges. Cystatins undergo frequent duplication events during evolution, and have a signal peptide and one or two disulphide bridges. The inhibitory consensus motif for C1A peptidases is formed by a conserved glycine residue in the N-terminal region, a QxVxG motif in the central region of the polypeptide and a tryptophan in the C-terminal region [17], although proteins with variants in these motives are also active as inhibitors, such as the sialostatins from ticks that lack the conserved tryptophan residue [18]. The inhibitory activity against C13 peptidases is achieved by an asparagine included in the consensus motif S/T-N-D/S-M/I/L based on vertebrate and plant cystatins [19,20], although the ability to inhibit legumains by variants of this motif has not been tested. Some studies have suggested that cystatins from arthropods have functions related to the control of endogenous proteolysis, the balance of host–vector immune relationships, innate immunity, or antimicrobial defence [21-24]. In addition, two other types of cysteine peptidase inhibitors, the propeptide regions of C1A peptidases (I29) and the thyropins (I31) have also been reported in arthropods [25,26]. However, with the exception of the cystatins that contributes to blood feeding in ticks by suppressing host immune response [27], their potential role in the feeding process of arthropods has yet to be investigated.

Comparative genomic analyses provide valuable insights into the conservation and evolution of protein families, which could aid to elucidate issues concerning their function [28]. Thus, we have performed a comparative study of the gene families of cysteine peptidases and their inhibitors in representative species of different taxonomic groups belonging to Arthropoda. This analysis has been focused in those cysteine peptidases potentially involved in *T. urticae* feeding [4]. Results indicate mite-specific proliferations of C1A and C13 cysteine peptidases and their inhibitors I25B cystatins, as well as a correlation between the *in silico* expression of specific cystatins with putative digestive cysteine peptidases, providing evidences for their involvement in the feeding process of the spider mite.

## Results

### C1A and C13 cysteine peptidases and their inhibitors in fully sequenced arthropods

As previously described, a proliferation of both C1A and C13 cysteine peptidases was detected in the genome of the two-spotted spider mite [1]. To obtain further insights on the genomic content of proteins putatively related to the proteolityc digestive process of *T. urticae*, we extended the search of members from these peptidase families and their inhibitors to other species of arthropods, whose genomes have been completely sequenced and annotated, and drafts of these sequences are available on the web. The selected species were: two acari, *T. urticae* and *Ixodes scapularis* (black legged tick); one crustacean, *Daphnia pulex* (common water flea); and ten insect species, the dipterans *Drosophila melanogaster* (fruit fly) and *Anopheles gambiae* (African malaria mosquito), the lepidopteran *Bombyx mori* (domestic silkworm), the coleopteran *Tribolium castaneum* (red flour beetle), the hymenopterans *Camponotus floridanus* (carpenter ant), *Apis mellifera* (honey bee) and *Nasonia vitripennis* (jewel wasp), the hemipterans *Rhodnius prolixus* (kissing bug) and *Acyrthosiphon pisum* (pea aphid), and the phtirapteran *Pediculus humanus corporis* (human body louse).

**Figure 1:**
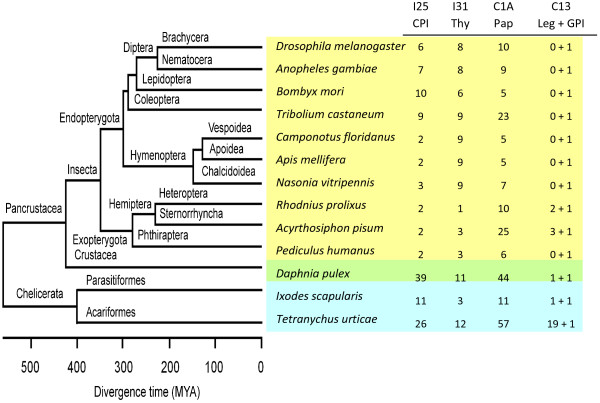
**Number of cysteine peptidases and their inhibitors in selected arthropod species.** Schematic evolutionary tree of fully sequenced arthropods including for each species the number of inhibitory cystatin (I25CPI) and thyropin (I31Thy) domains and the C1A (papain-like, C1APap) and C13 (legumain-like, C13Leg and GPI:protein transamidases, C13GPI) cysteine peptidases, putative targets of both inhibitors. MYA, million years ago.

The results obtained from genome extensive searches compared with the location of each species in the phylogenetic tree of arthropods are summarized in Figure 1. All the species have C1A cysteine peptidases, although the highest number of genes was found in *T. urticae* when compared with all the insect and even the other acari species analysed. Likewise, from the C13 family of peptidases, a strong proliferation of legumains was detected in *T. urticae*, 19 genes, against a maximum relative to the three genes encoding legumains in the herbivore pea aphid. On the contrary, a unique gene encoding a C13 GPI:protein transamidase was present in all species analysed. Differences in the number of cysteine peptidase genes could be hypothetically correlated with differences in the number of their putative inhibitors. Thus, we look for the gene content of both, the cystatins (I25CPI), which are putative inhibitors of C1A and C13 peptidases, and thyropins (I31Thy), which are putative inhibitors of C1A cysteine peptidases. As for C1A papains, the number of cystatins was considerably higher in *T. urticae* and in the crustacean *D. pulex* than in other insect species, whereas the number of thyropins was only slightly higher. Interestingly, *I. scapularis*, the other chelicerata species used in this study, showed a similar number of both cysteine peptidases and inhibitors than the insect species analyzed. As clade-specific proliferations were previously detected for C1A cysteine peptidases and C13 legumains [1], next analyses were focused to the evolutionary features of cysteine peptidase inhibitors.

### Gene content evolution of I25 cystatins in arthropods

**Figure 2:**
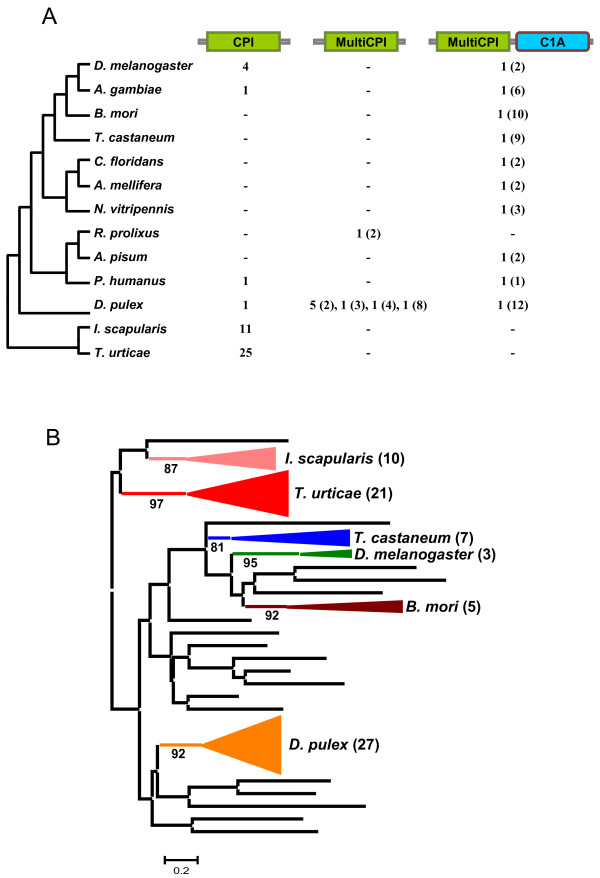
**Evolutionary features of cystatins in arthropods.** (**A**) Number of cystatin domains in each species distributed in base of their protein architecture. In brackets the number of repeats in multicystatin domains. CPI, single cystatin domain; MultiCPI, multicystatin domains. (**B**) Phylogenetic tree using the selected cystatin sequences from the different arthropod species. Coloured triangles show species-specific gene proliferations. In brackets the number of proteins in each subtree.

Arthropod members of the cystatin superfamily belong to the stefin and cystatin lineages. Stefins were not present in the insect species and one stefin encoding gene was found in the genomes of the mite *T. urticae* and in the crustacean *D. pulex*. Architectures for proteins containing domains of the cystatin lineage vary among different clades. Three different architectures were detected (Figure 2A). Proteins including a unique cystatin domain were only detected in the annotated genomes of the two species of acari analysed, the crustacean, the dipteran species, and in *P. humanus*. Multicystatin proteins containing only cystatin domains, whose number range from two to eight, were only present in *D. pulex* and *R. prolixus*. In contrast, multicystatin proteins (two to twelve domains) containing an additional C1A cysteine peptidase domain were found in the crustacean *D. pulex* and in all the insect species analysed, with the exception of *R. prolixus*. Interestingly, these multicystatin-C1A peptidase proteins were not present in the acari species.

To understand how the cystatin lineage has evolved in the different arthropod clades, the individual I25B domains were aligned by MUSCLE (see Additional file 1A). Extensive amino acid differences avoid the construction of a robust phylogenetic tree using all the cystatin sequences. Thus, sequences contributing to extensive gaps in the conserved regions of the alignment were discarded and a phylogenetic tree constructed by the maximum likelihood PhyML method (see Additional file 2A). The corresponding schematic cladogram is shown in Figure 2B. As highlighted, clado-specific proliferations are detected, supported by approximate likelihood-ratio test values (aLRT) higher than 80%. This cladogram suggests that the evolution of the cystatin family in arthropods is the result of extensive duplications from the ancestral genes probably determined for specific features in each clade.

### Gene content evolution of I31 thyropins in arthropods

**Figure 3:**
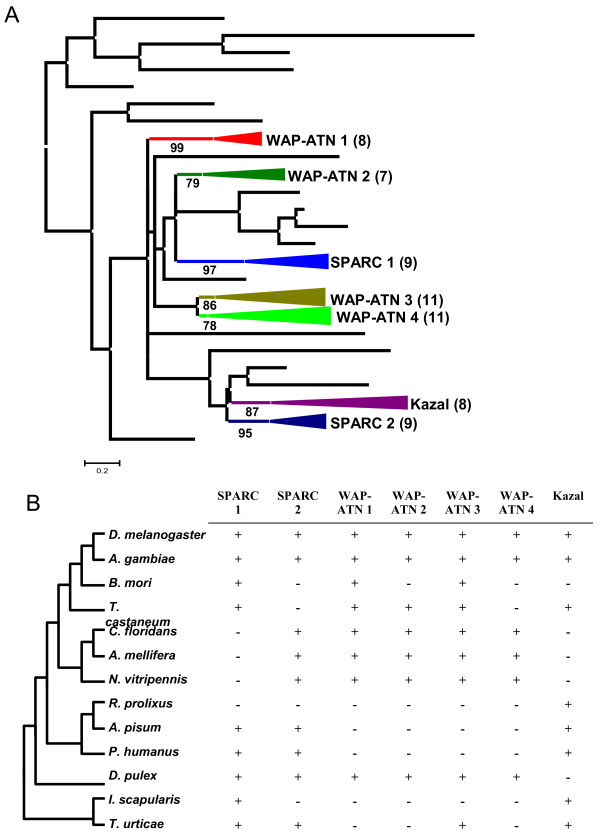
**Evolutionary features of thyropins in arthropods.** (**A**) Phylogenetic tree using the selected thyropin sequences from the different arthropod species. Coloured triangles show thyropin gene proliferations associated to other protein domains. In brackets the number of proteins in each subtree. (**B**) Presence (+) or absence (−) of thyropins members of each subtree in the different arthropod species. WAP, whey acidic protein; ATN, antistasin; SPARC, secreted protein acidic and rich in cysteine.

As for the cystatin family, we performed a phylogenetic analysis of the individual thyropin domains to know how this family has evolved in the different arthropod clades. The I31 domains were aligned by MUSCLE (see Additional file 1B), and a phylogenetic tree was constructed by the maximum likelihood PhyML method (see Additional file 2B), after discarding sequences contributing to extensive gaps in the conserved regions of the alignment. The corresponding schematic cladogram is shown in Figure 3A. A search for additional domains in proteins containing thyropin regions gives a broad combination of different domains (see Additional file 3). The repertoire of additional domains includes the serine peptidase inhibitory domains Kazal 2, Kunitz and Antistasin (Ant), the peptidase related inhibitory domain WAP (whey acidic protein), the calcium binding domain SPARC (secreted protein acidic and rich in cysteine), and the putative GTP-binding domain nuc121. As remarked, groups including thyropin domains associated to a second peptide domain and supported by approximate likelihood-ratio test values (aLRT) around or higher than 80% were found. When we analysed the composition of these groups, we observed that they are composed by proteins belonging to different arthropod species (Figure 3B). Proteins with thyropin domains associated to SPARC, WAP-ATN or Kazal domains are shared by insect, crustacean and acari species. A comparison of the cladograms showed in Figures 2B and 3A implies a different evolutionary process from cystatins and thyropins, with a small weight of clade-specific proliferations in the evolution of thyropins.

### Structural features of *T. urticae* cystatins

**Figure 4:**
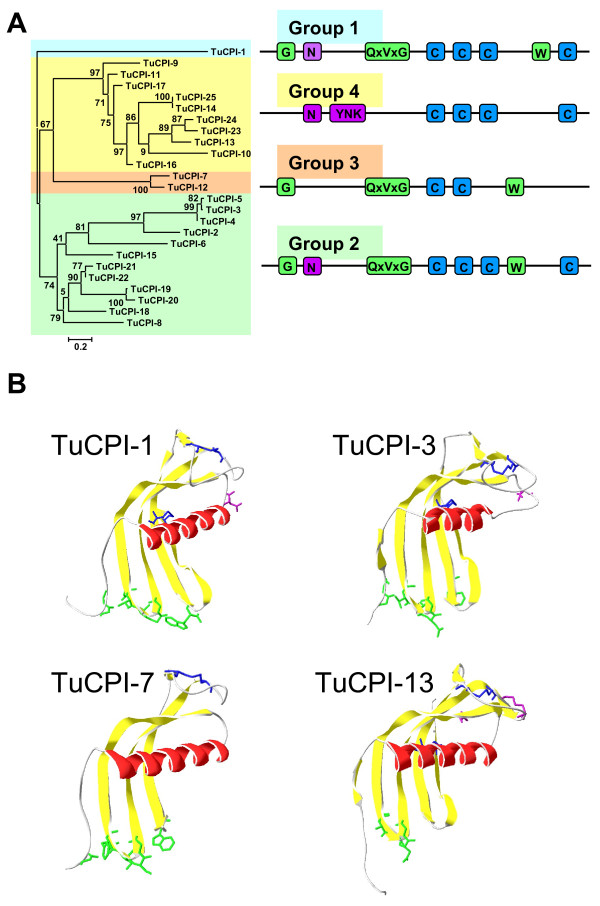
**Evolutionary features of cystatins in*T. urticae*.** (**A**) Phylogram of *T. urticae* cystatin sequences showing the presence of functional conserved residues in the four cystatin groups deduced from the phylogenetic tree. Amino acid sequences responsible of the inhibitor-C1A enzyme interaction are shaded in green. Putative sequences involved in C13 legumain inhibition are coloured in purple. Cysteine residues involved in disulphide bridges are in blue. (**B**) Homology models of *T. urticae* cystatins created using SWISS-MODEL. The residues in the region that interacts with C1A peptidases are coloured in green. The residues that could be involved in legumain inhibition are coloured in purple. Cysteines are coloured in blue. Red, α-helix; yellow, β-sheets.

Since the cystatin lineage had a clade-specific pattern of proliferations, they could be related to the inhibition of the expanded groups of C1A and C13 cysteine peptidases of *T. urticae*. Thus, we analysed in more detail the sequence-structure relationships in this family of proteins. To find how spider mite cystatins are grouped, their I25B domains were aligned by MUSCLE (see Additional file 1C), and a phylogenetic tree was constructed by the maximum likelihood PhyML method. Additionally, a representation of the alignment was done including the location of the key residues involved in cysteine peptidase inhibition, and of the secondary structures predicted in their tri-dimensional conformation (see Additional file 4). Four different groups were detected, which had specific features in their amino acid sequences (Figure 4A) and a different number of protein members. Whereas groups 1 and 3 consisted of 1 or 2 genes, groups 2 and 4 have been extensively expanded in restricted scaffold regions probably due to recent gene duplication events. Group 1 is formed by TuCPI-1, which is the only *T. urticae* cystatin sequence that have the consensus motifs for C1A peptidase inhibition: a G in the amino acid part of the protein, the conserved QxVxG in the first loop and a W in the second loop. Besides, it has the consensus motif including the asparagine responsible to legumain inhibition as well as the four cysteine residues involved in disulphide bridges. Group 2 is constituted by cystatins TuCPI-2 to −6, -8, -15, and −18 to −22. Most members of this group retain the asparagine putatively involved in C13 inhibition surrounded by variants of the consensus inhibitory sequence, and all conserved motifs for C1A inhibition with the exception of the tryptophan in the second loop, which is replaced by several different amino acids. In contrast, most of these proteins have a conserved tryptophan residue four amino acids before the expected location of this. Group 3 is composed by TuCPI-7 and −12, and its members are similar to that of the second group, with the new conserved tryptophan residue, but they lack the cysteine residues that form the second disulphide bridge and the asparagine important for legumain inhibition. Finally, group 4 including TuCPI-9 to −11, -13, -14, -16, -17, and −23 to −25, is the most striking clade. The proteins that belong to this group do not present the conserved residues involved in C1A inhibition, neither the G nor the QxVxG or any of the tryptophans in the C-terminal part of the molecule. However, they maintain the four cysteine residues and have several conserved motifs in their sequences, as an YNK motif after the putative α-helix location. Besides, some of its members have an asparagine residue in the location where it is involved in legumain inhibition.

To determine how amino acid differences can influence the tri-dimensional structure of the cystatins, the structures of the TuCPI-1, -3, -7, and −13 proteins, representatives of each group, were modelled using the crystallographic structure of the cystatins from chicken egg (1YVBI), soft tick (3L0R), and human cystatin F (2CH9) (Figure 4B). TuCPI-1 and −7 aligned to chicken egg cystatin at sequence identities of 30% and 23%, and with Q-MEAN Z-scores of −3.36 and −2.87, respectively. TuCPI-3 and −13 aligned to soft tick cystatin and human cystatin F at sequence identities of 28% and 22%, and with Q-MEAN Z-scores of −2.32 and −1.76, respectively. These results imply relatively accurate models for *T. urticae* cystatins. From models, strong differences in the putative region for C1A peptidase inhibition were observed. The consensus amino acid residues in TuCPI-1 fit with a canonical interaction with C1A peptidases. On the contrary, the conserved tryptophan in TuCPI-3 and −7 located at the end of the third β-sheet could be involved in a distinct interaction with these peptidases leading to changes in their inhibitory specificity. In the case of TuCPI-13, the lack of conserved residues in the domain responsible to C1A peptidase inhibition could mean a lack of inhibition to these peptidases. TuCPI-1 and −3 also have an asparagine in the loop after the conserved α-helix that could be involved in legumain inhibition. This asparagine was absent in TuCPI-7 and −13. However, four group cystatins have at least two different conserved motifs (YNK and SKPY) at the spatial region where the C13 inhibitory activity is achieved. A role for the asparagine of the YNK motif in legumain inhibition could be hypothesized, although the conserved motifs could be alternatively related to a different function.

### Expression profiling of cysteine peptidases and inhibitors

**Figure 5:**
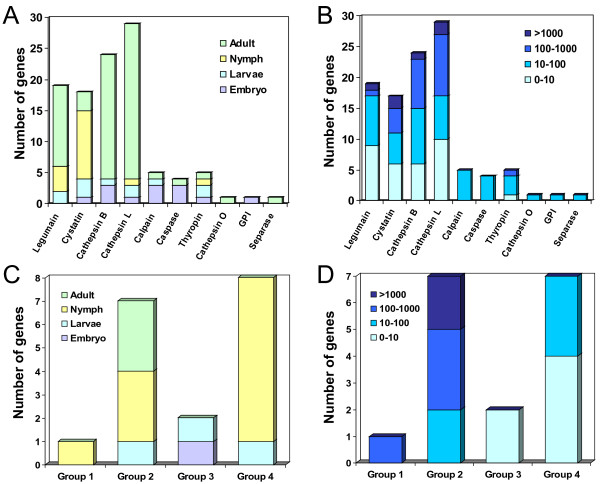
**Expression profiling of cysteine peptidases and their inhibitors in*T. urticae*.** (**A**) Number of genes for the different cysteine peptidase and inhibitor groups assigned to the developmental stage (embryo, larvae, nymph and adult) in which their highest expression was detected. (**B**) Number of genes for the different cysteine peptidase and inhibitor groups assigned to the intervals of normalized expression values (0–10, 10–100, 100–1000, >1000) in which their highest expression was detected, independently of the mite developmental stage analysed. (**C**) Number of genes for the different cystatin groups (Groups 1 to 4, see Figure 4) assigned to the developmental stage in which their highest expression was detected. (**D**) Number of genes for the different cystatin groups assigned to the intervals of expression values in which their highest expression was detected, independently of the mite developmental stage analysed.

To correlate genomic proliferations to gene expression, an *in silico* analysis of transcriptome expression was performed using the RNA-seq information available at the BOGAS *T. urticae* database. Most genes for cysteine peptidases and inhibitors had transcriptomic data. Only the stefin and seven cystatin genes had not RNA-seq information available. Figure 5A shows the developmental mite stages in which the highest level of expression was detected for each gene analysed. Genes belonging to the C1A cathepsin L and B, C13 legumain and I25 cystatin groups were more expressed in the feeding stages of development, mainly in the adult phase. These genes had a wide expression range, having many genes expression values over 100 and even over 1000, but also lesser than 10 (Figure 5B). Furthermore, the comparison of the expression levels between adults and embryos showed that more than 75% of the cathepsin B-like, cathepsin L-like, legumain and cystatin genes with an expression value higher than 5 were significantly overexpressed in adults (Table 1). On the contrary, genes included in the C1A cathepsin O, C13 GPI:protein transamidase, C2 calpain, C14 caspase, C50 separase and I31 thyropin groups, did not show a specific developmental pattern (Figure 5A), having most of them a similar level of expression in all the mite stages analysed (data not shown), with normalised expression values among 10 and 100 (Figure 5B). Likewise, none of the genes for these families was significantly more expressed in adults than in embryos (Table 1).

**Table 1:** Expression of cysteine peptidases and inhibitors in adults against embryos

**Peptidase/Inhibitor**	**Adult> > Embryo**^**a**^	**Adult = Embryo**	**Embryo> > Adult**^**a**^	**Low expression**^**b**^	**Total**
Legumain	9	1	0	9	19
Cystatin	6	1	0	11	18
Cathepsin B	15	4	0	5	24
Cathepsin L	15	4	1	9	29
Calpain	0	5	0	0	5
Caspase	0	4	0	0	4
Thyropin	0	4	1	0	5
Cathepsin O	0	1	0	0	1
GPI	0	1	0	0	1
Separase	0	1	0	0	1

^a^ Number of genes with a FDR value ≤ 0.05 and a fold change ≥ 2.

^b^ Number of genes expressed with a value ≤ 5 in adults and embryos.

A similar analysis was conducted for the four groups of spider mite cystatins. The different groups have specific expression patterns (Figure 5C, D). TuCPI-1, the only protein in group 1, was highly expressed in all developmental stages (data not shown) with its maximum expression in nymphs. Members of group 2 showed the highest expression values and were most abundant in adults and nymphs. Conversely, group 3 cystatins presented the lower level of expression, with one gene mostly expressed in embryos and the other in larvae. Finally, the members of group 4 had, in general, low expression values, but had an interesting developmental pattern, with a weaker expression in adults or embryos than in nymphs.

## Discussion

Cysteine peptidases are crucial in different arthropod physiological processes [11-14], including the digestion of dietary proteins in mites and some insect species [8-10]. The dominant cysteine peptidase activity detected in body extracts of *T. urticae* points out their main role in proteolytic digestion after feeding [4,5]. In addition, the proliferation of C1A papain and C13 legumain families in the recently annotated genome of *T. urticae* and changes in expression detected in these peptidase genes after host change suggest their implication in the feeding process and, most probably, on the ability of mites to feed on the large number of plant hosts [1].

The broad multigene family of papain-like cysteine peptidases and the unusual proliferation of legumain peptidases in *T. urticae* were found to have no counterparts after extending the analysis to the annotated genomes of arthropods available so far, including ten insect species belonging to different orders, the crustacean *D. pulex*, and the acari *I. scapularis.* Gene expansions in C1A cysteine peptidases were also found, though in a lower extent, in insects such as the aphid *A. pisum* and the beetle *T. castaneum*, which also rely on cysteine peptidases for proteolytic digestion [29,30]. On the contrary, none of the insects analyzed belonging to the orders Diptera (*D. melanogaster* and *A. gambiae*), Lepidoptera (*B. mori*), Hymenoptera (*C. floridanusA. mellifera* and *N. vitripennis*) and Phthiraptera (*P. humanus*), with a digestive system mostly based on serine peptidases [31], presented this extensive proliferation of cysteine peptidases. Hemipterans (*R. prolixus*) and ticks (*I. scapularis*), that in addition to cysteine peptidases use serine and/or aspartyl peptidases for proteolytic digestion [31], did not also have an expansion of their cysteine peptidases. In the case of the crustacean *D. pulex*, in which digestion commonly relies on trypsins and chymotrypsins [32], proliferation of cysteine peptidase genes could be associated to specific adaptations to the lifestyle of a planktonic filter feeder in a highly variable aquatic environment. Thus, we could conclude that, in general, extensive C1A cysteine peptidase duplications in arthropods are correlated to their corresponding diet and could be related to nutritional functions.

Large-scaled gene amplification for legumains is a striking feature restricted to *T. urticae*. Legumains, also called asparaginyl endopeptidases, have been involved in the degradation of host hemoglobin in ticks [9,33]. A legumain from *Ixodes ricinus* is located intracellularly in the vacuoles of gut epithelial cells where digestion occur throughout the whole duration of feeding [15], and contributes directly to the cleavage of hemoglobin and/or the processing of other gut peptidase zymogens [34]. In most acariform mites, digestion is considered to be composed of an extracellular phase achieved by enzymes secreted to the lumen of the midgut, followed by an intracellular one that can be performed in free floating cells [2,3,35]. Legumains could be involved in the intracellular phase by processing some other peptidases or by a direct action on the plant feed proteins. Large expansion of this family in *T. urticae* could be related to the broad range of C1A cysteine peptidases that need to be activated for digestion or to their direct role on host selectivity by processing plant toxic proteins.

Massive gene expression data have been previously used to detect different peptidases in the gut of insects [30,36-38] and ticks [39]. Since digestive proteolytic enzymes should be abundantly generated to deal with the higher volume of food that must be hydrolysed in free living developmental stages, genes involved in the proteolytic digestion are expected to be expressed at higher rate in free leaving stages comparing to embryos. Transcriptomic data analysis in *T. urticae* indicates that most cathepsin B and L peptidases and legumains are expressed at a higher rate in larvae, nymphs and adults, confirming their putative role in proteolytic digestion. On the contrary, other gene cysteine peptidase families like caspases, separases, calpains and GPI:protein transamidases have similar levels of expression in the four developmental stages analysed, supporting a role for these genes in some other endogenous processes across the whole life of the spider mite.

The activity of this extended number of peptidases must be regulated in the acari. Among cysteine peptidase inhibitors, the cystatin, thyropin and C1A cysteine peptidase propeptide families have been previously described in arthropods. Cysteine peptidase propeptides are inhibitory domains that are included in all the C1A peptidases analysed in this study. They have a conserved role in the control of C1A peptidase activity before the inhibitory domain is released and the peptidase becomes active [40]. The *T. urticae* genome does not contain small propeptide-like genes similar to those present in *Bombyx*[22].

Remarkably, the expansion in peptidase genes of the families C1A and C13 in *T. urticae* is accompanied by a proliferation of cystatin inhibitors putatively targeting both enzyme families. The physiological functions of this extended number of I25 cystatins in *T. urticae* may be related to their inhibitory activity on specific C1A and C13 peptidases. In insects, cystatins have been associated to processes related to the regulation of endogenous protease activity, such as insect morphogenesis and development [23,41], and/or in the inhibition of heterologous cysteine peptidases, e.g. during insect immune response and plant feeding [21,42]. Moreover, cystatins have been previously related to blood-feeding in ticks [27,43,44], where they are expressed in salivary glands and the midgut contributing to suppress host immune response. The fact that cystatins have evolved differentially in arthropod clades implies a specific function for the members of the proliferated groups in each clade. Thus, besides their potential implications in some other physiological processes, spider mite cystatins could have a role in mite feeding by regulating their own digestive cysteine peptidases, by inhibiting cysteine peptidases of the host after feeding, and/or by contributing to counteract host defence mechanisms. Several results support the putative involvement of cystatins in mite feeding: i) transcriptomic data analysis indicate that group two and four cystatins are consistently detected at greater levels in larvae, nymphs and adults relative to embryos; ii) the amino acid sequences for several spider mite cystatin groups have diverged deeply from consensus cystatin motifs related to cysteine peptidase inhibition. In fact, group 4 cystatin genes code for a new type of cystatins with no known paralogs in living organisms that putatively contain C13 peptidase binding domains, but do not contain canonical C1A peptidase binding domains. These results support an evolutionary scenario in which cystatin family may have evolved in the spider mite to control the proliferation of divergent C1A cysteine peptidases and C13 legumains involved in protein digestion. Alternatively, cystatins in *T. urticae* may have evolved as specific adaptations to the lifestyle of an extremely polyphagous species to deal with highly variable resources.

On the contrary, limited data on the physiological roles for thyropins are available. Thyropins are thyroglobulin domains capable of exhibiting inhibitory activity, which has been reported mainly against C1A cysteine peptidases [45]. No arthropod thyropins have been characterized to date, although some members are present in the sialotranscriptome of several ticks [46,47]. The possible implication of thyropins in the control of peptidases involved in feeding comes from the reduction of cysteine peptidase activity and deleterious effects on larval growth when the thyropin equistatin isolated from sea anemone *Actinia equine* was introduced in the diet of the insects *T. castaneum* and *Leptinotarsa decemlineata*[48,49]. However, the parallel evolution of thyropins, with complex architectures shared by different arthropod clades, and their transcriptional pattern, having most of them a similar level of expression in adults and embryos, suggest a common conserved role that could be in some cases related to feeding, but no specific for the spider mite nutritional aspects.

## Conclusions

Comparative genomic analyses have provided valuable insights into the conservation and evolution of cysteine peptidases and their inhibitors in *T. urticae*. A phylogenetic analysis of these gene families in representative species of different arthropod taxonomic groups has allowed us to state that clade-specific proliferations are common to C1A papain-like and C13 legumain-like peptidases, as well as to the I25 cystatins, whereas the I31 thyropins are evolutionarily more conserved among arthropod clades. Extensive duplications and transcriptomic data for spider mite C1A and C13 peptidases support their role in proteolytic digestion. The expansion of the I25 cystatin family of inhibitors and their highest expression in feeding stages suggest a role for some cystatin members in mite feeding by regulating endogenous or exogenous peptidases and/or by contributing to counteract host defence mechanisms. In conclusion, mite-specific proliferations of both peptidases and their inhibitors are in accordance with mite’s feeding features and support a key role for these proteins in allowing the broad plant host feeding range described for the two-spotted spider mite.

## Methods

### Sequence searches

Blast searches for cystatins, thyropins and cysteine peptidases were performed in publicly available genome databases. Sequences for *Tetranychus urticae* were obtained at the BOGAS (Bioinformatics Online Genome Annotation System) website [50]. Sequences for other arthopods were identified by searching the current genome releases at: the ant Fourmidable database [51]; the AphidBase [52]; the invertebrate vectors for human pathogens VectorBase [53]; the *Daphnia* wFleaBase [54]; the FlyBase [55]; the *Bombyx mori* SilkDB [56]; the BeeBase [57]; the wasp NasoniaBase [58]; and the BeetleBase [59]. Blast searches were made in a recurrent way. First, a complete amino acid arthropod sequence from data banks corresponding to a protein of the family was used. Then, the protein sequences of each arthropod species were used to search in the species. Finally, after an alignment of the proteins found in arthropods, the conserved region surrounding the catalytic sites from the species most related was used to a final search in each arthropod species.

Information about gene models for all these proteins is compiled in Additional file 5.

### Domain architecture prediction

Amino acid sequences for arthropod proteins putatively including at least one cystatin or thyropin domain were subjected to a sequence search in the Pfam database v 26.0 [60] to know the combination of domains within each protein. From these results, the domain architecture of each protein was manually schematized.

### Protein alignments and Phylogenetic trees

Alignments of the amino acid sequences were performed using the default parameters of MUSCLE version 3.8 [61]. Sequences with extensive gaps were manually excluded from phylogenetic analysis using the multiple alignment editor Jalview version 2.7 [62]. Phylogenetic and molecular evolutionary analyses were conducted using the programs PhyML 3.0 and MEGA version 5.0 [63,64]. The program PROTTEST (2.4) was employed for selecting the model of protein evolution that fits better to each alignment according to the corrected Akaike Information Criterion [65]. The parameters of the selected models were employed to reconstruct the displayed clan CD cysteine peptidases trees by means of a maximum likelihood PhyML method using a BIONJ starting tree. The approximate likelihood-ratio test (aLRT) based on a Shimodaira-Hasegawa-like procedure was used as statistical test for non-parametric branch support [66]. All families were also analysed with the Maximum parsimony and the Neighbour-Joining algorithms, and with different gap penalties. No significant differences in the tree topologies were detected.

### Molecular modelling of *T. urticae* cystatins

The three-dimensional structures of the *T. urticae* cystatins were modelled using the standard automated routine of SWISS-MODEL program [67]. The known crystal structures of the cystatins from chicken egg (PDB identifier 1YVBI), soft tick (3L0R), and human cystatin F (2CH9) were used to construct the homology-based models. The template structures were selected on the basis of highest sequence similarities. Models were evaluated with the QMEAN Z-score for predicting the absolute quality of a model [68]. The Swiss-PdbViewer program [69] was used to generate the single images of protein models.

### *In silico* transcriptome expression

The transcriptomic information available at the BOGAS *T. urticae* website [50] was used to the developmental expression analyses. The protocol to normalized read counts of RNA-seq Illumina reads has been previously described [1]. To determine significant differences in the levels of gene expression between spider mite embryos and adults, we defined as differentially expressed genes that for which the false discovery rate (FDR) corrected p-value was ≤ 0.05 and for which the fold change was ≥ 2 (either up- or down-regulated).

## Competing interests

The authors declare that they have no competing interests.

## Authors’ contributions

MES, PH-C, VG and MM carried out the sequence recovery and analysis. ID, FO, MG and MM designed the study and carried out the interpretation of the results. MM drafted the manuscript. All authors read and approved the final manuscript.

## Supplementary Material

Additional file 1 **Alignments performed using the MUSCLE program of the amino acid sequences corresponding to the proteins used in this study.** (A) Alignment of cystatin domains from selected arthropod species. (B) Alignment of thyropin domains from selected arthropod species. (C) Alignment of *T. urticae* cystatin domains. Click here for file

Additional file 2 Complete phylogenetic trees of the cystatin (A) and thyropins (B) domains from selected arthropod species. Click here for file

Additional file 3 Domain architectures of the proteins with thyropin domains from the selected arthropod species. Click here for file

Additional file 4 Alignment of the different four groups of cystatin sequences from *T. urticae* showing conserved motifs and structural features. Click here for file

Additional file 5 Information about gene models and accession numbers corresponding to the proteins (cystatins, thyropins, C1A peptidases and C13 peptidases) used in this study. Click here for file
