# Supplementary material for: Cysteine peptidases and their inhibitors in Tetranychus urticae: a comparative genomic approach
Source: BMC Genomics. 2012 Jul 11;13:307. doi: 10.1186/1471-2164-13-307 (PMC3407033; doi:10.1186/1471-2164-13-307)
Supplement: Additional file 1 — Alignments performed using the MUSCLE program of the amino acid sequences corresponding to the proteins used in this study. (A) Alignment of cystatin domains from selected arthropod species. (B) Alignment of thyropin domains from selected arthropod species. (C) Alignment of T. urticae cystatin domains. [file 1471-2164-13-307-S1.pdf]

Additional file 1. addfile1.doc

Alignments performed using the MUSCLE program of the amino acid sequences corresponding to the proteins used in this study. A. Alignment of cystatin domains from selected arthropod species. B. Alignment of thyropin domains from selected arthropod species. C. Alignment of *T. urticae* cystatin domains. Ag, *Anopheles gambiae*; Am, *Apis mellifera*; Ap, *Acyrtosiphon pisum*; Bm, *Bombyx mori*; Cf, *Camponotus floridanus*; Dm, *Drosophila melanogaster*; Dp, *Daphnia pulex*; Is, *Ixodes scpularis*; Nv, *Nasonia vitripennis;* Ph, *Pediculus humanus*; Rp, *Rhodnius prolixus*; Tc, *Tribolium castaneum*; Tu, *Tetranychus urticae*.

A

```
BmCPI-1h        -E------------TVSG---ISIRRLIKSSIKELEK------NPDQKYKLIHLGTPYLV
BmCPI-1g        ---------QTL--DDGDYIDEELRNYYAERANQYLNQVS---DTNNLYKLITVHAIKYG
CfCPI-1a        -G-----CPYEL--NPNL---PSLLVFAEQALKSIDE-QT---ANDYKHKLMSIVKVTRS
AmCPI-1b        ---------PNL---------PGFISFGEQVAKSMDE-LI---QNDFKHKVIDIVKVTRA
NvCPI-1b        ---------------------PSLPGLSAFGNQVMQS-MDEAGVSDFKHKLISIVRVTRA
DpCPI-1d        -GVKTARSLSPL--SPDD---EEVKDIAAFALNRLDSFDD---SNSKKRILVTVVEGTAS
BmCPI-1i        -L-----NSANV--SAND---LEIRELVKQSLDKLEM-AS---VHRYKQRVIQINSFSTK
BmCPI-1c        -N-----IRKTF--EIDD---YKVSEMLQESLMYLDV-KS---NRNNKQKIVDVNSVSTQ
DpCPI-1a        IN-----ASEDV--ELKD---PTLVAISTFAANSILK-DAETFGIQNTSSLHKIMDAHVV
TuCPI-12        -G-----PWVTV--PVDD---PIIQKYTEQALERQNKEFG-----DRYKRLVSINQAKRQ
TuCPI-7         -G-----PWVPV--PVDD---PIIQKYAEEAVETRNKKYD-----GHYKRLMSVDVAKKQ
TuCPI-6         -G-----EWGRV--SMDN---PTVLELAELAVDDHNK-LS---TNDYYFKLVKITSVSFQ
TuCPI-2         -G-----GWRDV--DVDN---QTVHLLSQMAINHRNS-DE---DTLYYRKLVNVESARMQ
TuCPI-5         -S-----GWQTS--DANS---GTIKDLAQVATEHRNS-QI---NSLYYRTLVEIKSAKQQ
TuCPI-3         -G-----GWQTS--DANS---ETIKDLAQVATEHRNS-QI---NSLYYRTLVEIKSAKQQ
TuCPI-4         -G-----GWGSV--DANS---ETIKDLAQVATEHRNS-QI---NSLYYRTLVEIKSAKQQ
TuCPI-10        -T-----EWTPL--PSDD---PTVVKFANLAVADING-K----EKLFYNKLIQIKEAKSS
TuCPI-13        -T-----EWKPL--PVDD---PTVIKLANQAVAHINE-KW---EKLFYNKLIEIKEAKSV
TuCPI-23        -T-----EWTPL--PVDD---PTVIKLAKQAVADVNSQ-----EKFTYNKLIVITEAKSV
TuCPI-24        -T-----EWTPL--PVDD---PTVIKLAKQAVANVNSQ-----EKFNYNKLIEIQEPKSV
TuCPI-14        -V-----KYKPL--PVDD---STVIKLANQAVAKINA-EG---NGKFYNKLIEIKEARSK
TuCPI-25        -V-----KYKPL--PVDD---STVIKLANQAVAKINA-EG---NGKFYNKLIEIKEARSK
TuCPI-9         -P-----EWKPL--PVDD---PTVIKLADLAVVDINA-KE---NSSYYNKLVQIRAAKSR
TuCPI-16        -I-----EWTPL--PVDD---PTVITLADQAVAYINA-QD---NSLYYNKLIQIKEAKSR
TuCPI-11        -G-----PWTPL--PVDD---PTVIKLADQAVVDINA-QD---NSLYYNKLILIRDKRRV
TuCPI-17        -G-----PWTPL--PVDD---PSVIKLAKQAVVDINA-HD---KSLYYNKLIEIREAKSR
TuCPI-8         -S-----VWKSI--PVDD---PTVNLLTEKGIEHRNK-ND---NSIYYEKLIAIKKAQTQ
TuCPI-15        -G-----GWNSL--STDD---STVNQLAIKSVNHHNS-VN---NSAYYKKLVKIQEARYQ
TuCPI-20        -G-----AWSSN--SVDD---PLVAELAAKGLDYENR-YG---NSFYYKKLITIKEARIQ
TuCPI-19        -G-----AWSSN--SVDD---PLVVELAAKGLDYENR-YG---NSFNYKKLITIKEARAQ
TuCPI-18        -G-----GWKIL--PVDD---PTVVQLAAKGVECYNK-NS---NNIYYNKLIKIKKAASE
TuCPI-22        -G-----GWSSI--SVDH---PTVIQLAAKGVEHHNK-IA---NNLYYKKLITIKEAKSQ
TuCPI-21        -G-----GWSSL--SVDH---PTVIQLAAKGVEHHNK-IA---NNLYYKRLISIEEAKSQ
TcCPI-1f        -R-----GESPV--AKDN---EYVIKYLEAALNQLDA-ES---EHENKFKVHEFISATSQ
TcCPI-1h        -G-----GESPV--AKDN---EYVIKYLEAALNQLDA-ES---EHENKFKVHEFISATSQ
TcCPI-1d        -G-----GESPV--TKDN---EYVVKYLEAALNQLDS-ES---PHENKFKVHEFISATSQ
AgCPI-2g        -G-----SSNEL--TAEELKDKSHVERIRAGMVSYNS------ERSKAYNEFEILAGSTQ
AgCPI-1         -G-----GVSDD-PELNK---EEHAERIGAALAT---------TDGHAGKAYKLHRVTKQ
AgCPI-2d        -G-----AAQEL--TPEEYAKEEHQTRIRTGLQQQSA-LV---DGSGNERKVKVVGATVQ
AgCPI-2e        -G-----CATPL--AANEYGNSEHQERIDKILSF---------HGLTRGNSLKVINATSQ
AgCPI-2f        -G-----GSRSL--SQDELAAAEHLERVDKILVSS--------GGSKESSNARIVSGTVQ
DmCPI-1b        -G-----RHKPY----DE---EAAKAQLQKSLDKLTA------GEGPHYKIVKVYSASRQ
NvCPI-1e        -G-----APTSH--SKDD---PEVQRYVQLGLEKYTE-NY---QGTNQPMISNIKDVSVQ
AmCPI-1d        --------------DINN---PTVQELANKGLKKFSE-NS---EGSNEPMIVEIVDASRQ
CfCPI-1b        -G-----GTITT--SVND---PEVQLYANKALRKVSE-ES---DGPNEPFIVEIIEASVQ
DmCPI-5         -G-----APKVL--EGED--LASAQQTLEASLTKLAA------GEGPHYRLSKILSATSQ
DmCPI-2         -G-----APKPL--DGDD--LSKAKELLDTTLAKLAT------GDGPNYQVVNVISASSQ
DmCPI-3         -G-----GVSQL--EGNS--RKEALELLDATLAQLAT------GDGPSYKAINVTSVTGQ
DmCPI-4         -G-----GISQL--EGNE--RKEALELLDATLAQLAN------GDGPSYKALNVTSVTGQ
AgCPI-2c        -G-----GATPV--DVKE---PTHIERVRLGLVGYES---------GKHSNFEILFGTVQ
TcCPI-1c        -G-----APNRI--DKNS---EKIRQFVKEGINGFNANYN---SKNNKVKPVEVVSATTQ
TcCPI-1i        -G-----GATEI--DKKS---DKVKQYVRESLTHLNT-QL---TSSNKVKPVEVLSATSQ
```

```
TcCPI-1e        -G-----GITEV--DKDD---DEVKTFVREGLLNLNT-HL---TTSNKVKPVEVVSASVQ
TcCPI-1g        -G-----GIKEV--DKNN---DEVKTFVHEGLLNLNT-HL---TTSNKVKPVEVVSASVQ
TuCPI-1         -G-----GWMPK--DIDY---EPAKDNAKYAAKLIND-QS---NDMYFQNLIHIHDVKSQ
ApCPI-1a        -G-----GYNDV--EADS---EKIRELALFSLDSITQ-QT---MSKRSLGLIRVVSAKSQ
IsCPI-9         -G-----GWEKR--DPHE--NPHFQELAHYAVSKGSK------PKRYYDTVVTLIEVYTQ
IsCPI-10        -G-----GWKTQ--DLTN---PKFENLAHYAVSTQVE------GREYYDTVLGILEVKTQ
IsCPI-4         -G-----GYTRKTDHQTN---PKYLELAHFATSSWSAGQA---NKAYYDTVEEVLEAQTQ
IsCPI-3         -G-----GYSLKTDHHTN---PKYLDLAHFATSSWSAQQP---GKTHFDTVEEVERVETQ
IsCPI-1         -G-----GYSERANHQAN---PEFLNLAHYATSTWSAQQP---GKTHFDTVAEVLKVETQ
IsCPI-2         -G-----GYRER-SNQDD---PEYLELAHYATSTWSAQQP---GKTHFDTVVEVLKVETQ
IsCPI-5         -G-----GWRSR--DVYS--DPAYAELAHYAVSSQAG------DSEFYDTVLELLEVETQ
IsCPI-11        -G-----VWRRHHPDMD----PRYKEWAHFAISSQVE------DRTNFDTLMTLISVESQ
IsCPI-7         -G-----VWIKHQPDMD----PRYKEWAHFAISSQVE------DRTNFDTLMNLMSVESQ
IsCPI-6         -G-----VWRKHHPDVD----PRYKEWAHFAISSQVE------DRTNFDTLMTLISVESQ
IsCPI-8         -G-----VWRRHHPDVDH----RYKEWAHFAISSQVE------DRTNFDTLMTLISVESQ
ApCPI-1b        -G-----AKISL--NSND---KKVQDIVAYALLSIDR-QE---GSNKPHVLSKIINVSKQ
PhCPI-2a        -G-----DRNLL--NISD---PEIIRLSKIALLKLNEEKN---IFNQEKILVKILKASKQ
PhCPI-1a        -G-----GEKET--DVNN---PTVLSAIKSTMVKLNENLS---SGENEKKFVETLKATVQ
BmCPI-1f        -G-----GKHEE--DPSD---KEFKVLAQESLHEYAR-LE---KNDFIHKVIDVNRVSTQ
BmCPI-1d        -G-----GLQLQ--DAHD---QKYKLLAEESLRQFLQ-KN---GTTKPHTVVRLNKVTTQ
BmCPI-1e        -S-----GMTES--DVTE---PHYKKLAQQSLNQFLK-ES---GNTKPHIVVRLNKVTTQ
RpCPI-1         -G-----EPKEA--SVES---EEIKAAANFAAERIDQ-MS---NSIYKQILVRILEATSQ
DpCPI-1f        -G-----GHGQV--DTMS---TEIEAYSDFALAVIEE-QS---NADEKLKVTKILASSVQ
DpCPI-1l        -G-----GISPA--DPSS---EEIKAHAAFALQAIQA-QS---NSRNLLNIVRIKNAGTQ
DpCPI-1e        -G-----AQQSA--DKSS---PYIQSVADFATKAISQ-RS---NG-NILNLIRVIRADTQ
DpCPI-1c        -G-----CPANM--DVDS---IQVKELANFALSALED-AA---NCTKVQSILRITKATSQ
DpCPI-1k        -G-----GVTSM--DLHS---QKIKELSDFAVSAISL-RS---NEPNAPSKVRVLNASKQ
DpCPI-1j        LG-----TYISI--DVSD---EQVGELATFATTIL-S-HV---RNAGDLTLVKISSASKQ
DpCPI-8c        -N-----KFSSI--DVDD---PDVKAVGEFAMKVANT-AAASSGHSAPVKLVKILKAEWQ
DpCPI-10b       -G-----GFTSV--SIND---ADVLEMARFATNALSV------NKASPLVLVAVVQAEKQ
DpCPI-5b        -G-----GYSAA--SASE---EDVQEIAKFATHALSQ-NA---NQASPFVLVQVVKAEKQ
DpCPI-1g        -G-----GYSAA--DPTD---PAILEIANFATQAASSSQSTDASNSAPFTLAKIHSAKKQ
DpCPI-6b        -G-----GYVIR--NVND---SDVKEMAAFAFSIL-T-AN---SHPHHLALIKILKAESQ
DpCPI-9a        -G-----GFTPA--KLND---AEIVKMVDFATTAV-S-AS---MNSGPVKLLKIVKAEIQ
DpCPI-1i        -G-----GYCPI--DPNE---KAVKEMANFAAISL-S-RS---MNSVPLKLAKIRFAERQ
DpCPI-1h        -G-----GYSQS--DIND---ASVKEMADFATQAI-S-RS---TNAGALSVAKIISAETQ
DpCPI-6a        -G-----GYKPA--MVDA---ADVKRMAAFATSAI-S-AS---DSGPAVQLIWIRRAWKQ
DpCPI-8a        -V-----GFYRI--DVDD---VEVKEIALFAAKALSK------SQNSILKLNKIILAEAE
DpCPI-8e        -D-----GYSPV--DVDN---SYVKEIAAYATTAI-S-SS---RNSITLSLQRILNAEAR
DpCPI-8h        -N-----GFSPV--DVNN---PEVKEMANFATSSI-S-AS---DNPFILKLIKVVDAEAK
DpCPI-8g        -D-----GFASL--DVED---VKVKEIAAFASNSISA-------NSGPVTLLRILKAEAQ
DpCPI-8f        -S-----GFSPA--DVQA---DDVREMAYFATHVIS-------NTRNPVALAEIVKAESQ
DpCPI-4a        -G-----EFSPI--DVND---SEVREIADFATTAI-S-AS---SNAGPFRLIKILKAESQ
DpCPI-3         -G-----GYSPA--NVHD---IDVNEMADFSRRAI-S-SR---SNSGPSTLIRIVKAEKQ
DpCPI-4c        -G-----GFMPM--NVNN---EQVREIAEFATSAI-S-SK---INSGPVTLVNIVMAESQ
DpCPI-7b        -G-----GYKTI--DVND---ATVKEMAEFATSAI-P-EK---MNSGPVTLIKIIKAKSQ
DpCPI-2d        -G-----GFSTI--DVDD---AYIKEIADFATTTI-S-AN---NNSGPVRLIRIIKAESQ
DpCPI-7a        -G-----GFSPL--DVEN---VTVKEMAAFATTAI-S-AN---TNSGPVTLVKVVKAQSQ
DpCPI-9b        -G-----GFSSL--DVDD---AEVKEIAAFASSAI-S-AN---TNSGPLTLVKVLKAQSQ
DpCPI-4b        -G-----GYSPI--DVND---AKVKEIARFAAAAV-S-IN---LNSGPIALVNIVKAESE
DpCPI-8c2       -G-----GFSSI--DVND---PAVKEIASFATSTIAS-KK---SKSGPTKLIKIVKAESE
DpCPI-2a        -G-----GFSPI--DVDD---PNVKEIADFATSTI-S-ES---SNSGPLSLIKIVKAESQ
DpCPI-2b        -G-----GFSPI--DTDD---ASVKEMADFATTRI-S-ES---SNSGPLALIKVVKAESQ
DpCPI-8b        -G-----GFSQI--DVDN----DVKEIAIFAGTAI-A-SK---RNSGPARVTKIAKAESQ
DpCPI-8d        -G-----GFYPT--DVEN---PEVKEMAIFASKVL-E-SK---RNSGPTKVTKIVTAESQ


BmCPI-1h        P-SLDSDVP---IKLSFLI----GSTNCTKEVDIENSPLQCFLDGSKSS--KPCTSFV--
BmCPI-1g        K-Q--MGRN--IVQMYIEV----APTFCLRHADENELGGCEEIE-ALDH--KLCYGRL--
CfCPI-1a        VPVSSNMIQ---YQLLLLI----GESECLKNALEQEQECPLRA--TNSI--KLCSVTF--
AmCPI-1b        IPPSSNIIQ---YQILLHI----GESDCLKNAIEQSECSVQL---NSSF--KICLVTF--
NvCPI-1b        V-P--PGANVVQYQLLVEI----GESNCLRTSLIEIAECPLQS--NLPI--KLCLVTF--
DpCPI-1d        T----EGRS-KTFKMKIHV----ALADCPEGGSANNEACLASLSGNPQH--YLCDIQV--
BmCPI-1i        I-T--TGKV---TTIDFDV----GYTSCLK-YEWVDDVMTCQFLEHLPR--RHCVSKV--
BmCPI-1c        I-N--AGLL---TEIIFTV----AYTSCRNDVKVDINTCNVLE--DEPL--RNCKAQI--
DpCPI-1a        PASKPSPTQ---YMLTIEL-----GNNCTNTNDNC---------TGGR--HICEVSV--
TuCPI-12        T-L--SGYR---FEIEMII----RETDCNKNDPKKHQCQFNS---ARAP--EPVVFDV--
TuCPI-7         F-I--SGYR---FEIEMTI----RETECHQNDPKKHQCQFNS---ARAP--EPVVFDV--
TuCPI-6         A-L--NGIK---YSITFII----GQTKCFKTDPNHKKCDLLH---KNINIMNLCSYLF--
TuCPI-2         V-V--SGLK---YEVTLVI----GETHCAKEDEAAMLCQVEP---NSLR--EKCVYTF--
TuCPI-5         V-V--NGMK---YELTLVL----ADTNCAKQDAGAKLCPVGQ---GAAK--EECVYTI--
TuCPI-3         V-V--NGMK---YELTLVL----ADTNCAKQDAGAKLCPVGQ---GAAK--EECVYTI--
TuCPI-4         V-V--NGMK---YELTLVL----ADTNCAKQDAGAKLCPVGQ---GAAK--EECVYTI--
TuCPI-10        -----KAKF---YVFKVIV----GKTDCPIRGPYTDACQIKD---DAPR--KECSVYY--
TuCPI-13        A-D--DGIT---YMLKMII----RLTYCPKSKPYHDDCEIRQ---ASSA--QICTVEG--
TuCPI-23        V-D--DGVT---YMLKMII----QKTYCPLSKPYHDDCEINQ---GLPG--STCIVDA--
TuCPI-24        V-D--DGVT---YMLKFII----RSTYCPISKPYHDDCEIR----ELPG--RICSVDA--
```

```
TuCPI-14     LRH--SKIT---YTIKVIL----RKTYSHKSKPYTDACGIND---TAPP--KLCTIDA--
TuCPI-25     LRH--SKIT---YTIKVIL----RKTYSHKSKPYTDACGIND---TAPP--KLCTIDA--
TuCPI-9      L-M--SGIE---YELRLDI----QKTDCPKSKPYTDACQINV---TTLP--PICTVHL--
TuCPI-16     V-A--DKVE---YELKLVI----RITDCPKSKPYTDACQINQ---DEPP--KLCTIDA--
TuCPI-11     G----DKIE---YELKLVI----RQTDCPKSKPYTDTCQINQ---DTLP--QICTYEL--
TuCPI-17     V-A--DKIE---YELKLVI----RITDCPKSKPYTDACQINQ---DLPP--KLCTYEL--
TuCPI-8      A-L--APAK---YKIEFLI----GPTECLKTDPNSASCQIST---NKAS--ETCIFVF--
TuCPI-15     V-V--AGFK---YEIKFLI----GKTECAKTGNYTDSCQVAV---NSPT--ELCTYVF--
TuCPI-20     A-AKPYGVN---HEVKLLI----GQTDCAKWNVNATSCEVSP---NAIP--ELCTYVT--
TuCPI-19     A-DKVSGIN---HEVKLLI----GQTDCAKWNANATSCEVSP---NATP--ELCTYVI--
TuCPI-18     I-V--AGML---YEIKFLI----GATDCVKSEPDASSCKVSP---NAIP--KLCTYHF--
TuCPI-22     V-V--AGMN---YEVKFLI----GKTECVKSDANAASCEVSA---NAIP--ELCTYVF--
TuCPI-21     V-V--AGIN---YEVKFLI----GKTECVKSDANAASCEVSA---NAIP--ELCTYVF--
TcCPI-1f     I-V--SGHI---YRINAKV----ILSDCKKTVSTERGQCGTLK--DAKP--KTCKFEV--
TcCPI-1h     I-V--SGHI---YRIKAKV----ILSDCKKSISTERGQCGTLK--DAKP--KTCKFEV--
TcCPI-1d     T-V--SGHI---YRIKTKV----VLSDCKKTASTERGQCGTLK--DAKP--KTCKFEV--
AgCPI-2g     Q-V--AGSL---YKYTFRV-------------------------TSESD--IVCKISI--
AgCPI-1      V-V--SGVQ---YVYFISF--------------------------ENEESGQQYKITV--
AgCPI-2d     L-V--AGKS---YTYRLSF--------------------------PDDELKRVCKLTV--
AgCPI-2e     V-V--AGMK---YVYFIQH----------------------------NN--AVCKLTS--
AgCPI-2f     I-V--SGKL---YKYAVEF--------------------------DVDGSSKLCKLSS--
DmCPI-1b     V-D--SGIL---TRIDADL-------------------------IDGSEEQHRCIVDI--
NvCPI-1e     V-V--SGLL---YKIQTDI----GVSTCSKGTV-TGDCQLSK---DHGV--EECVIEA--
AmCPI-1d     V-V--SGYL---YKIRVKL----GTSNCPKGTK-----EKCQLKEGTEI--KECLFSI--
CfCPI-1b     V-V--AGKL---YKIKAKL----GTSDCPKGTKTNCQLQA-----GSEV--KECLITV--
DmCPI-5      V-V--SGFK-NDYSVELID----NQ--------------------GAT---KVCQVDI--
DmCPI-2      L-V--AGSL---YKFEVKL--------------------------SNGAETKECNVKI--
DmCPI-3      V-V--AGSL-NTYEVELDN--------------------------GSDK--KQCTVKI--
DmCPI-4      V-V--AGRL---NTYEVQL----DN--------------------GSEI--KQGTVQI--
AgCPI-2c     V-V--AGTI---HRYKIAL----KDDD------------------QKVY--STCDVKVFT
TcCPI-1c     V-V--AGTL---YKITTKI----SESDCSKNDNKDLDDCNILE--GASP--KTCELEV--
TcCPI-1i     V-V--AGTI---HRIKVKI----SESDCSKDDEKDFDECNIRE--GASP--KICEVKV--
TcCPI-1e     V-V--AGSL---HRIKVKI----SESDCSRNDQKDFEQCNVLE--GASP--KLCEMEV--
TcCPI-1g     V-V--AGSL---HRIKVKI----SESDCSRNDQKDFEQCNVLE--GASP--KLCEMEV--
TuCPI-1      V-V--GGVK---YNITFDM----SKTICRKNEIDSDKPEQCVPDRNATI--KRCYAVV--
ApCPI-1a     V-V--AGIN---YKIKLLV----CEKDSTLGENIVMDPKNC----------RSCDITI--
IsCPI-9      L-V--AGVN---YRLNYTY----ATTDCRTDQEYKPSKCRPK---GKVR--GWCESIV--
IsCPI-10     I-V--DGVM---FMLKFTT----TQSTCKIEAGVEYSKLNCHPRTSKVV--LALHGVV--
IsCPI-4      V-V--AGIN---YKLTLKV----AESVCEITSQYTKEACTPKP--DAVR--KTCTTVI--
IsCPI-3      V-V--AGTN---YRMTLKV----VESVCELTSTYSKEACTAKA--NAAH--RNCITVI--
IsCPI-1      V-V--AGTN---YRLTLKV----AESTCELTSTYNKDTCLPKA--DAAH--RTCTTVV--
IsCPI-2      T-V--AGTN---YRLTLKV----AESTCELTSTYNKDTCQANA--NAAQ--RTCTTVI--
IsCPI-5      V-V--AGMN---YRLKFST----AETACKVGVD-EYSRERCLPKVNLPK--ATCTAVV--
IsCPI-11     V-TRPQDVT---SLETSSK----EQHAAPKRTSIVLVTFLVSFCFLQPY--MLCTAVV--
IsCPI-7      V-I--VGVD---YKLKMKV----AESDCVIGVD-SYSRERCYLKVDVPY--MLCTAVV--
IsCPI-6      V-I--AGVD---YKLKMKV----AESNCVIGVD-SYSRERCHLKVDAPY--MICTALV--
IsCPI-8      V-I--AGVD---YKLKMKV----AESDCVIGVD-LYSRERCHLKVDVPY--MICTAVV--
ApCPI-1b     I-V--SGII---YNIELEI--------CDNSTSEVDEKKC-----------RICNIKV--
PhCPI-2a     I-I--SGSL---TELTLQI----LE--------------------KNVP--KYYVAKI--
PhCPI-1a     V-V--SGTL---TRVLLRI--------------------------NQGEETHYCYSKV--
BmCPI-1f     T-V--SGKI---YNIHFSA----VPTSCSTAVQDPSFCEQKD---GSSI--LQCHARI--
BmCPI-1d     V-V--SGTL---IRLDFVA----APTG------------------EESR--YQCHSEI--
BmCPI-1e     V-V--SGTV---TQLDFVAAPTG----------------------EESH--YQCHSKI--
RpCPI-1      V-A--AGIK---MDLKLEL----GNTECMKNMDKKANCEVVSE--NAEK--MICRVSV--
DpCPI-1f     I-V--QGKN---IRLSLEV----ASTSCKKDQPIGDNCAIDE---SKGF--QVCNIQI--
DpCPI-1l     T-V--AGKK---IYLTIEI----GQTKCPANET-SQSCSFDD---QTDR--QLCKIEI--
DpCPI-1e     L-V--AGKK---VTLDVEV----GFTNCSKAEGAGTFCQLDS---SQAN--VICHVAV--
DpCPI-1c     V-V--SGTL---YVLTIEL----VDTNCIRSENTDRSQCPANELTEGNH--RQCTVGI--
DpCPI-1k     V-V--SGMM---YTLQLEL----NFVDCQQDSEACIRR-------------QICNVSI--
DpCPI-1j     V-L--DGPN---YRLGLQV------------SSV-----------DGTN--LMCDVVV--
DpCPI-8c     V-VDAVGRN---FKLTLEL------------DDG-----------AEES--LLCVVSV--
DpCPI-10b    I-V--AGVN---YRLQLKF------------NGQQLESE------ENHF--IDCQVTV--
DpCPI-5b     I-V--SGIN---YRLHVEL---------KENAD------------SANV--ISCTVVV--
DpCPI-1g     V-V--AGIN---FKLDLEF--------------------------TRLNESLFCRVIV--
DpCPI-6b     V-V--AGTN---YKMALLF------------ANRPQHH-------SRYL--LLCDVIV--
DpCPI-9a     A-V--SGTK---YKLNLEL------------AGA-----------YSKV--IPCEVVV--
DpCPI-1i     V-V--AGFN---YRLDLEF------------TE------------PRGT--VHCKVVV--
DpCPI-1h     V-V--SGRN---YKITLQV------------QG------------DAGV--QTCTVVV--
DpCPI-6a     V-V--SGTN---YKLILEL------------LNTN----------TGQV--LLCEVIV--
DpCPI-8a     D-V--AGKN---FKLVLRL-------------ENLDEEEVSF---SKSF--INCEVVV--
DpCPI-8e     V-F--GGTN---YKLTLEL------------DHFIAGA-------KAEN--LLCKVIV--
DpCPI-8h     F-L--FGKN---FKLILRV------------KNMVKGAD------GDRE--MLCEVVV--
DpCPI-8g     T-V--AGKN---YKLIIEL-------------------------IGTERDIQICDVVV--
DpCPI-8f     AHA--AGRN---YKLTLKL------------DSMVKETTAGIPTFQSGD--LLCEIIV--
DpCPI-4a     VGI--GAVN---FKLTLEV------------DGA-----------DEKN--LRCEVVV--
DpCPI-3      V-V--AGMN---YKLTLEM------------ENA-----------NDGV--ILCDVIV--
DpCPI-4c     T-V--AGKN---YKLTLEL------------EGS-----------QRDK--HLCKILA--
```

```
DpCPI-7b        F-V--AGIN---FKLTLEL------------EG------------VQGA--IQCDVIV--
DpCPI-2d        I-V--AGKN---FKLTLKL------------NSAID---------EADS--LLCDVVV--
DpCPI-7a        V-V--AGLN---YKLTLEL------------NG------------AEGA--ILCEVTV--
DpCPI-9b        V-V--AGLN---FELRLEL------------KG------------AKGA--ILCEVLF--
DpCPI-4b        A-V--AGRN---YKLILEL----EGS-------------------DGEA--RICEIVV--
DpCPI-8c2       N-V--AGIN---YKLTLEL----NQPLAIDRS-------------------IICDVIV--
DpCPI-2a        V-V--AGRN---YKLILEL----SSVVDGAS--------------ETEE--TLCEVIV--
DpCPI-2b        V-V--AGMN---YKLTLEL----GSAADGAV--------------GS-N--LICNVLV--
DpCPI-8b        I-V--AGTN---YKLTLEL----NQPLA-----------------TEKF--LICDALI--
DpCPI-8d        A-V--AGTN---YKLTLEI----SQPIAVVE--------------TERF--LLCKVVV--


BmCPI-1h        -------WFVP-NT-KDI-----YQI-NVQC
BmCPI-1g        -------WPSP----DDELVV--QSV-SVIC
CfCPI-1a        -------EQRP-WLPTSL-----KII-RNNC
AmCPI-1b        -------EEKP-WQQSSR-----KIV-KNNC
NvCPI-1b        -------EERP-WQSGSR-----KIT-RNNC
DpCPI-1d        -------LVPL----RDSRFVQRRLV-NSRC
BmCPI-1i        -------FERL-WA-ANG-----KNI-DVSC
BmCPI-1c        -------WDRT-WI-EDG-----TQI-KVSC
DpCPI-1a        -------LDAP-WN-EKRVL---DED-KTKC
TuCPI-12        -------WVKG----D---------------
TuCPI-7         -------WVNT----RNQ-------------
TuCPI-6         -------WIKP-GTPKTV-----EII-HHAC
TuCPI-2         -------WLEA-KT-QNT-----NIV-TSSC
TuCPI-5         -------WIES-TK-EAP-----EVT-SSSC
TuCPI-3         -------WVES-TK-EEP-----AVT-SSSC
TuCPI-4         -------WIES-TK-ETP-----EVT-SSSC
TuCPI-10        -------YYNT-VEWKHG---------TYTC
TuCPI-13        -------HKPS-GS-EEI-----KIS-NLSC
TuCPI-23        -------HKPV-GS-EEI-----KIG-RLQC
TuCPI-24        -------HKPV-GS-EEI-----KIG-RLQC
TuCPI-14        -------YVRV-GS-DES-----KIV-ILRC
TuCPI-25        -------YVRV-GS-DES-----KIV-ILRC
TuCPI-9         -------HEDP-GS-KEI-----KIT-AFQC
TuCPI-16        -------YVRA-GS-EEN-----KIY-KFLC
TuCPI-11        -------FSRA-GS--KN-----KIT-TLQC
TuCPI-17        -------FVRA-GS--KN-----KFT-TLQC
TuCPI-8         -------LIRR-GS-NDI-----HIT-RDFC
TuCPI-15        -------WMPP--V-DKD-----RIT-SFDC
TuCPI-20        -------WVSP-DL-HWR-----ELT-QASC
TuCPI-19        -------WVSP-DL-HWR-----SLM-QASC
TuCPI-18        -------WIKS-WS-GFE-----QIT-QVSC
TuCPI-22        -------WVRP-GS-DNA-----QIT-QASC
TuCPI-21        -------WVQP-GS-DNA-----QIT-QASC
TcCPI-1f        -------FEQL-WVPNSR-----QI--KTDC
TcCPI-1h        -------FEQL-WVPNSR-----QI--KTEC
TcCPI-1d        -------FEQT-WVPNSR-----RI--KTDC
AgCPI-2g        -------WERV-WL-E---SQDQRKY-NVKC
AgCPI-1         -------WERP-WL-KEKDPAEARKI-TFEV
AgCPI-2d        -------WEKP-WL-KEKAPQEAFKA-SFEC
AgCPI-2e        -------WERV-WL-AQSHPEDAYKY-TYDC
AgCPI-2f        -------WERP-WL-EKK-----DPTEAYKY
DmCPI-1b        -------WTKV-WVRKDE-----HEI-TFKC
NvCPI-1e        -------WSQP-WL-DKG-----NPKITVKC
AmCPI-1d        -------WSQP-WI-DKG-----SPKITINC
CfCPI-1b        -------WSRP-WI-DHG-----SPEITITC
DmCPI-5         -------WSQS-WL-PN------GIQVTFRC
DmCPI-2         -------WDRP-WL--HE-QGEATNV-KVQC
DmCPI-3         -------WTQP-WL--KE-NG--TNI-KIKC
DmCPI-4         -------WSRA-WLKENG-----TNI-KIKF
AgCPI-2c        PLPSAANGSKP------------DY-DFDC
TcCPI-1c        -------WEKL-WE-NFR-----QFT--IKC
TcCPI-1i        -------WDKP-WQ--NF-----RQY-NITC
TcCPI-1e        -------WDKP-WE-DFR-----RYT--IKC
TcCPI-1g        -------WDKP-WE-DFR-----RYT--IKC
TuCPI-1         -------YERP-WE-SKR-----QLL-DHKC
ApCPI-1a        -------WEQS-WL-NKK-----NVT-KVAC
IsCPI-9         -------YEMP-CE-HIV-----QIS-QHHC
IsCPI-10        -------ASRS--------------------
IsCPI-4         -------YEKV-WE-NMK-----SVS-SFSC
IsCPI-3         -------YENL--Q-GEK-----SVS-SFDC
IsCPI-1         -------FESL--Q-GDK-----SVS-SFEC
IsCPI-2         -------YRNL--Q-GEK-----SIS-SFEC
IsCPI-5         -------YERP-WQ-NHR-----EVT-SYEC
IsCPI-11        -------NYRP-WE-HKT-----SLK-SYNC
IsCPI-7         -------NYRP-WE-HKA-----SLK-SYNC
```

```
IsCPI-6         -------NYMP-WE-HKT-----SLK-SYNC
IsCPI-8         -------NYRP-WE-HKA-----SLK-SYNC
ApCPI-1b        -------WEQA-WE-NNK-----NTS-EFNC
PhCPI-2a        -------WERP-WL-NKT-----EVT-FFDY
PhCPI-1a        -------WEQL-WL-NKT-----EVL-AHHC
BmCPI-1f        -------WSRP-WL-GKK------TT-TITC
BmCPI-1d        -------WERP-WL-KKT-----DI--EVNC
BmCPI-1e        -------WEQP-WL-KKT-----SI--EVDC
RpCPI-1         -------WSQP-WK-QSSGKSHLKLS-KFYC
DpCPI-1f        -------WDRA-WL-QEK-----QVT-DLNC
DpCPI-1l        -------WTRP-WL-NER-----TVT-SLKC
DpCPI-1e        -------WDRA-WL-NDR-----KVT-NVTC
DpCPI-1c        -------WDQP-WL-NSK-----QIR-EPQC
DpCPI-1k        -------WEQP-WL-KKR-----EMT-KLTC
DpCPI-1j        -------YSQG----NAR-----QLT-YSSC
DpCPI-8c        -------FEQSTWKMIQL-----SFV-TREV
DpCPI-10b       -------FDQV-WT-ATR-----QIT-SFQC
DpCPI-5b        -------YDQS-WT-STR-----QIT-SSEC
DpCPI-1g        -------LEQS-WL-SVR-----EVT-NMTC
DpCPI-6b        -------FDQP-WT-HTR-----KLT-EYKC
DpCPI-9a        -------FHQP-KT-NTQ-----KML-RSSC
DpCPI-1i        -------FDQA-WT-STR-----ELS-QMQC
DpCPI-1h        -------YDQS-WT-KTR-----KLT-SFKC
DpCPI-6a        -------FDQP-WT-NTL-----ELR-SFRF
DpCPI-8a        -------FDQS-WT-STR-----ILR-ESDC
DpCPI-8e        -------FDQK--E-DYR-----KMT-DSLC
DpCPI-8h        -------FDQS-WT-STR-----KVT-ESTC
DpCPI-8g        -------FDQE-RS-QTR-----ILI-DSKC
DpCPI-8f        -------FFQT-WS-NTR-----ILS-ESNC
DpCPI-4a        -------FDQS-LV-KSW---------TSVC
DpCPI-3         -------FDQP-WT-NTR-----RLR-ESSC
DpCPI-4c        -------FDQP-WT-KTR-----ILS-EFNC
DpCPI-7b        -------FYQR-WS-KTR-----KLT-QSKC
DpCPI-2d        -------FDQS-WS-QTR-----QLK-QSNC
DpCPI-7a        -------FDQS-WT-NTR-----KLT-ESKC
DpCPI-9b        -------LRQC-WS-QIS-----AAPFRSDC
DpCPI-4b        -------FDQP-WT-NTR-----ILS-NSNC
DpCPI-8c2       -------FDQT-WT-STR-----ILS-ESHC
DpCPI-2a        -------FHQP-WT-QTR-----KLS-KSNC
DpCPI-2b        -------FHQS-WT-HTL-----ELK-ESNC
DpCPI-8b        -------FDQS-WT-KTR-----ILS-EHRC
DpCPI-8d        -------FNQP-WT-KTR-----ILA-EWNC
```

B

```
TuThy-5a        ----IQQREKAINLNGKSFCSNRAGILVK-------------TGFLFETSEYQP-ECDYE
TuThy-5d        ----------------IQEYEITTLGKPY-------------DKL----------LC-DL
TuThy-4b        ----------CWSELTRRNEELKFLKKKF-------------DFVVGY---ELP-EC-NL
TuThy-4c        ---SEIEQMRCNCIRDKDLLRQANDKTNW-------------SDY----------DC-DH
AgThy-5b        VNSRLATTMNCKCARARKLLIDSKSL-------------------------EVP-EC-CP
AgThy-5b        -----QISMRCECSRLAAKARTLLNSQYP-------------V--------LTS-RC-DS
AgThy-4a        ----------CIYDRSTCLHTMHLDKDLV-------------G--------WIP-KC-NL
TuThy-5c        ----------CEQLLQPLANLTSFLGRKG-------------IDAIGLNSM----QC-DL
AgThy-5a        --------CQTMLESSKCYTAQKQFDDQR-------------QAGLIGHLMQRP-EC-DG
TuThy-5b        ----------CSVALSRYTSDPDNYLDYQ-------------SSR------HL--HC-SE
TuThy-1a        ----SSDTTSCTYRRQMETNSNTRLPGR-----------------------LIP-EC-RP
DmThy-3e        --------VEQATLKPMELKTTRCRALSK-------------TAP------FPV-SC-DE
AgThy-3e        -----------EPIAKRLSASATRCQALQ-------------MAAS-----FPV-AC-DT
DpThy-4a        ----------------KKGGGGGGGGSGE----------------------LIP-EC-DH
DpThy-5a        --------TRCQKMRQTQLKKGGGGGSGE----------------------LIP-EC-DH
TcThy-2g        --------------------SNTRCEALG-------------G------------QC-DT
NvThy-2f        ----------SAEGKEINKVLGSRCQAMR-------------ERG------FVPAIC-DK
CfThy-1f        ---------LEMDERRAGKILGTRCQAMK-------------NKG------HVPTIC-DP
AmThy-2f        ---------LETDEKQIGKVLGTRCQAMK-------------DKG------HVPAIC-DR
AgThy-4b        -----RMTCACSRRRDKLEKEGRFDVTL---------------------------HC-TQ
TuThy-1b        --------CDCILQRHQITSEKPKRVGH-----------------------FTP-QC-EE
DpThy-3f        -------------SSTCTSPEENSSGPL-------------MMSI-----SPP-VC-TL
TuThy-2b        --------PNCSESRQIALKNSANFSHSK-------------T--------LIP-SCNNQ
DmThy-2a        -------CNACLEAVKFARRQQERDPGY-----------------------FVP-RC-RK
TuThy-2a        ----------CLLERSANLAVKLESDHQS-------------GKEI-----LIP-ECDLT
CeThy-2a        --------LDCASQRRKALK--RKTDGDA-------------RI-------YIP-TC-SP
ApThy-2a        -------NDSLLRVKHRGRCKGDPTRTPD-------------S--------YMP-RC-KA
IsThy-2a        ----CPDARRCWSQRSQALEQVRSGASGV----------------------FVP-DC-SA
DpThy-2b        -------ANDCYSDREAAQEEVDHGAKGM----------------------YVP-EC-TP
ApThy-2b        -QEEEAEVNDCLTDRQEALDDPSTSSHK-----------------------YIP-EC-TV
DmThy-2b        -------DSNCWMDQSVTLEEQGHGGKSV-------------L--------FVP-QC-LP
NvThy-3b        ----------CISDRRSVMEDQRQNSDRK-------------F--------YIP-EC-TP
AmThy-3b        ----------CLTDRRSVLEDERQHSQEK-------------F--------YVP-AC-TP
CfThy-3a        -----------MSDRRSVLEDQKQNSQEK-------------F--------YIP-QC-TS
TcThy-3b        ----DEDPSDCLSDRQTALNDGGQF--------------------------YVP-EC-TP
PhThy-2b        --DQEPEANDCFSDRQAVLAEQRSNNL------------------------YVP-EC-TP
AgThy-2b        --------SDCLSDRKYALDEQKYGTNAL----------------------YVP-EC-TP
AaThy-2b        ------AESDCLSDRAAALEEQRSGATAL----------------------YVP-EC-TA
IsThy-2b        ------EVQDCSSARRKALEAHRQAPKGR-------------I--------YVP-EC-GS
PhThy-2a        ----------CRTRQPCFRELKKAERNRE-------------NQE------FVP-TC-LK
DpThy-2a        ---------------SAGGLGKKSAPTGE-------------T--------FIP-EC-NE
AgThy-2a        ---RGSCKDVCIASRTYALQQRASSPYTV-------------K--------YVP-RC-RE
AaThy-2a        ------CKDVCHASRTYALQHRSSSPYGV-------------K--------FVP-RC-RE
TcThy-3a        ----------CHFEKARCVNKNLTLAKRGPCRQQKLCRDWEIYRHSNPDYKFHA-TC-RP
RpThy-1a        ----------CSDKQPCWADRNGRDPEGG-------------LV-------FIP-KC-LS
NvThy-3a        -------------------------GARI--------------------------VC-RP
CfThy-4a        ------ETPACFSARLTARLGAR------------------------------P-IC-QH
AmThy-3a        -------TPACFSARLTARPSAR------------------------------P-IC-RS
TuThy-4a        --QCAYGNTKCLQHRTEALKTSLNESEPN----------------------YIP-GC-NT
DpThy-3c        ----------CEMMKEIADGRKPAEPGYN-------------LIL------KNP-RC-TP
DmThy-3c        ---------ACQHLQAIQLHQSSELGIPA-------------RQM------AVA-QCDPN
AgThy-3c        ---------ACQHLQTIQLHQASELGVPP-------------KQK------YIA-QC-DI
CeThy-1a        ----RAPRTECERRRASASSSSIRGG-------------------------FVP-AC-TA
DpThy-3a        ----------CERQRVETVKRARALQMTD-------------NDV------SLP-SC-DP
NvThy-2a        ----------CEQLALAARRRSRALGPGS-------------PAQ------LVP-RCDNV
CfThy-1a        ----------CEQLALAAVRRSRALGAEG-------------LSQ------FVP-RCDNE
AmThy-2a        ----------CEQLAQAAVRRSRALGPRG-------------PAQ------FIP-KCNNE
TcThy-2a        ----------CQTLRMAASRRAKALGVEA-------------RSV------RMP-RC-NK
DmThy-3a        ---------ACQHLRRSESRRAKALEGSSV---------------------RVP-RC-QK
AgThy-3a        ----------CQHLRRAEARRAKSLGDSL-------------LQTV-----RIP-RC-TA
DmThy-3d        ---------QCERLKLKNNLAAQRTGHS--------------SVW------FQP-RCDPV
BmThy-2d        ---------PCERLREKNEAAALKYGKGT----------------------FIP-VC-DA
DpThy-3e        --------STCEELREKNLKMAEKFKKVV----------------------FTP-KCNKA
AgThy-3d        ---------QCERLRLKNAMAAKRAGQPN-------------TW-------FQP-RCDPE
TcThy-2f        -------LSQCERLREKNQRAAERYHKPT----------------------FMP-RCEAN
CfThy-1e        -------LSQCERLREKNLKRSQRLKQPT----------------------FLP-KCNSD
AmThy-2e        -------LSQCERLREKNLKTSQRLKQPT----------------------FLP-RCDSE
NvThy-2e        -------LSQCERLRERNLKTSQKYKQPS----------------------FLP-RCNPD
NvThy-2c        ----------CEYLRDFGERMEGTREGMA-------------LAI------PAP-QC-EE
CfThy-1c        ----------CEYLRDFNDRMEGTREGMS-------------LAI------PAP-QC-EQ
AmThy-2c        ----------CEYLREFNERMEGTREDMS-------------LAI------PPP-QC-EK
BmThy-2b        -------------LRDFDEKMEGTVDGMK-------------LAL------PAP-TC-QQ
TcThy-2c        ----------CEYLHDFSESMEGTREGMT-------------LAL------PSP-SC-DS
DpThy-3b        ----------CLQQQQIAQLLSLTEREGK-------------G--------YVP-QC-DE
```

```
BmThy-2a        ------NVTMCTQQKMLAELLVVSEREGK-------------G--------YVP-QC-AA
DmThy-3b        -CQQPGNVTSCHQAKALADILSINEREGR-------------G--------YVP-ECNGP
AgThy-3b        ---QPHNVTVCHQARMLSELLSVNEREGR-------------G--------YVP-QCDGP
TcThy-2b        -CTHPKNVTECLHQRALSEILAVSERAGR-------------G--------YVP-QC-SE
CfThy-1b        ----------CHRDRMLAEILSVSERQGR-------------G--------YVP-QC-RE
NvThy-2b        ---------ACHRNRMLAELLSISERQGR-------------G--------YVP-QC-SE
AmThy-2b        ---------ACHRDRVLVEMLSISERQGR-------------G--------YVP-QC-SE
DpThy-3d        ----------CQHMQMIMKYKARENGLPA-------------NRL------FIP-RCRPE
CeThy-3a        ------------LRHEPPCHKAKHDVDPH-------------LLGV-----FLP-RC-DL
CeThy-4a        ------------LRHEPPCHKAKHDVDPH-------------LLGV-----FLP-RC-DL
BmThy-2c        ----------CQQRRALALHTAAESGNPP-------------AWA------WVP-QC-TE
BmThy-2e        ------EEKISFKTKCQMLQAEIDNGSEG----------------------YRP-RC-LS
TuThy-3a        ----------CFDRKSKPCLIEKSQRKKV-------------YDE------FVP-KC-DK
HsThy-1a        ----------CQHEREHILGAAGATDPQR-------------PIPPGL---FVP-EC-DA
TcThy-2d        ----------CQHQNALSQHQAHESGVPA-------------GRV------YIP-QC-TP
NvThy-2d        ----------CQHARAVAEHGARESGEPA-------------RRS------YIP-RC-DA
CfThy-1d        ----------CQHARAVAEHAARESGEPA-------------RRI------YIP-RC-DT
AmThy-2d        ----------CQHARAVAEHAARESGEPA-------------RRI------YIP-RC-DT
IsThy-1a        ----------CDVNRDSFLSTIEWCACFN-------------KSRA-----YIP-QC-DE
BmThy-1a        ----------CLRRAARPCTALARAHPQP-------------HAGA-----YVP-SC-DA
DpThy-1a        ----------CFSNADRPCVAMRRRSKPG-------------LLGA-----YIP-TC-DS
NvThy-1         -----------FSKAERPCAAVRKRSSPE----------------------VAP-NC-DS
CfThy-2a        ----------CFYKAERPCAAVRRRSSPD----------------------SAP-SC-DS
AmThy-1a        ----------CFSKAERPCAAVRKRSSPD----------------------VAP-AC-DS
ApThy-1a        ---------RCFDKTDRPCTAIKRRISPN-------------ALGV-----YVP-AC-DA
PhThy-1a        --------CHCFDKTDRPCTAAKRRLSPD-------------MLGV-----FVP-DC-DN
DmThy-1a        ----------CFEKTDRPCAAVRRRIAGD-------------FAGA-----YAP-DC-DI
AaThy-1a        --------CRCFEKTDRPCAAVRRRLGTD-------------LNGSG----YAP-DC-DS
AgThy-1a        ----------CFEKTDRPCAAVRRRLGND-------------LSGS-----YAP-DC-DS
CqThy-1a        ----------CFEKTDRPCAAVRRRLGND-------------LSGSG----YAP-DC-DS
TcThy-1a        ----------CFQRTERPCAAVKRKITPE-------------LLGV-----YVP-DC-DN
                                                                        *

TuThy-5a        TGQ--YKPKQCK--------------------------------TSK-CYCVDPDSGEKT
TuThy-5d        YGN--FKANQCF-------------------------------ESGSRCFCVD-SNGNRI
TuThy-4b        DGS--YKAKQCD--------------------------------ETS-CYCVN-QKGERY
TuThy-4c        LGN--YNPIQCF---------------------------------GDSCFCVD-QNGLPI
AgThy-5b        NGN--YKRLACR--------------------------------RGE-CYCVD-EDGGQV
AgThy-5b        KGS--FDQLQCV--------------------------------DDM-CVCVDMHTGRPT
AgThy-4a        DGT--YAAKQCR----------------------------GDRLSGR-CFCYS-EDGKRI
TuThy-5c        DGN--FVHRQCT--------------------------------DSE-CSCVN-SVGENV
AgThy-5a        DGN--FQPVRCI--------------------------------PGQTCYCVD-EEGKRI
TuThy-5b        NGN--FASLQCH--------------------------------DQL-CFCADPSTGQQV
TuThy-1a        DGK--FASLQCH---------------------------GEAVGGGRFCQCWD-PEGNII
DmThy-3e        AGA--FRPLQCN---------------------------------GRSCWCVD-AAGNQL
AgThy-3e        AGS--FEPMQCN---------------------------------GDTCWCVD-AAGNQL
DpThy-4a        LGR--FQPIQCL--------------------------PAKSSSGQVSCWCVD-EAGNQV
DpThy-5a        LGR--FQPIQCL--------------------------PAKYSSGQVSCWCVD-EAGNQV
TcThy-2g        TGK--FLPTQCE--------------------------------EET-CWCVD-EAGNQL
NvThy-2f        YGR--FEPTQCA---------------------------------GETCWCVD-EAGNQL
CfThy-1f        QGR--FEPTQCA---------------------------------GDTCWCVD-EAGNQL
AmThy-2f        QGR--FEPMQCA---------------------------------GDTCWCVD-EAGNQL
AgThy-4b        NGN--YEELQCD--------------------------------SGL-CWCADELTGSVQ
TuThy-1b        NGH--FKKYQCH------------------------------ASTGH-CWCVHPTNGTQI
DpThy-3f        KGD--YAREQSQ---------------------------------GEFSWCVD-TTGQPI
TuThy-2b        DSF-LYNEVQCH------------------------------ELPGF-CFCVRPSSGQLI
DmThy-2a        DGN--FAAMQCY--------------------------------GNNGCWCSD-SQGRPI
TuThy-2a        KGL--YKAQQCH------------------------------KETGY-CWCVDVNNGKPI
CeThy-2a        KNSLLYDKVQCY--DV----------------------------SIY-CWCVDELSGEPK
ApThy-2a        DGT--YFRIQCH------------------------------KKEGY-CWCVT-PAGKVV
IsThy-2a        DGA--FVQVQCH------------------------------RLTGY-CWCVD-AQGKVL
DpThy-2b        DNK--YQRVQCH------------------------------KSAGY-CWCANDETGKPI
ApThy-2b        DGR--YKHVQCY------------------------------KSVGY-CWCAQEDTGKPI
DmThy-2b        DGR--YQRIQCYSSTS----------------------------TSY-CWCVNEDTGKSI
NvThy-3b        DGR--YHKVQCY--------------------------------SGY-CWCVYQDTGKPI
AmThy-3b        DGR--YHRVQCY--------------------------------SGY-CWCVYQDTGKPI
CfThy-3a        DGR--YHRVQCY--------------------------------SGY-CWCVYQDTGKPI
TcThy-3b        DGR--YKKIQCY------------------------------KAAGY-CFCVHEDTGKNI
PhThy-2b        DGR--YNRIQCY------------------------------KSTGY-CWCVNEDDGKPI
AgThy-2b        DGR--YQRVQCY------------------------------RSTGY-CWCVNEDTGKNI
AaThy-2b        DGR--YQRVQCY------------------------------LSTGY-CWCVHEDTGKNI
IsThy-2b        DGT--YAEAQCH--------------------------------TGY-CWCVNQRTGRPI
PhThy-2a        DGT--FSPMQCH------------------------------NETGF-CWCVT-PKGKLI
DpThy-2a        DGR--FAEIQCH------------------------------QGTGY-CWCVT-PDGKPI
AgThy-2a        DGT--YAPVQCI--------------------------------DGGGCWCVN-GQGKQL
AaThy-2a        DGT--YAPVQCL--------------------------------ESVGCWCVN-GQGKPL
TcThy-3a        DGS--YAAAQCH------------------------------PDTGF-CWCVT-PQGIPL
```

```
RpThy-1a        DGR--YAPVQCH------------------------------EATGY-CWCVT-PQGKPL
NvThy-3a        DGT--YAPVQCH------------------------------PQTSY-CWCVT-PQGRPI
CfThy-4a        DGT--YAPIQCH------------------------------IETGY-CWCVT-PQGRPL
AmThy-3a        DGT--YAPVQCH------------------------------EETGY-CWCVT-PQGRPL
TuThy-4a        FGD--YSNYQCF---------------------------------GKRCFCVD-ENGDRI
DpThy-3c        QGE--FEEEQCD-------------------------------KDGQ-CWCVD-EFGVEL
DmThy-3c        NGK--WNQVQCS-------------------------------PDGH-CWCVD-DQGKIL
AgThy-3c        DGS--FRTIQCG--------------------------------PGNVCWCVD-EFGNEK
CeThy-1a        SGD--FERVQCE-------------------------------TNGRQCFCVN-TQGIEV
DpThy-3a        IGD--YEPVQCD------------------------------PLTGN-CFCVD-ESGFEL
NvThy-2a        TGE--FERVQCD-------------------------------PSGG-CYCVD-EYGGEV
CfThy-1a        TGE--FERIQCN-------------------------------PQGRGCWCVD-EIGAEI
AmThy-2a        TGE--FERIQCD-------------------------------PREKQCWCVD-EIGVEI
TcThy-2a        SGG--FEPIQCD------------------------------NEIVSSCWCVD-EAGFEL
DmThy-3a        NGD--FDAIQCQ-----------------------------DEKHGRDCWCVD-DYGVEL
AgThy-3a        LGD--FEPVQCS-----------------------------NELNGTECWCVD-EYGVEI
DmThy-3d        TGH--WSPVQCL--GKQPQPMDRHTEIVSRAFASEPAASAGEEAPGV-CWCAD-KKGAPL
BmThy-2d        SGA--WEPVQCM------------------------------SHIDV-CWCVS-ARGEPL
DpThy-3e        NGD--WEPVQCL------------------------------EEVGI-CWCVD-KDGEHI
AgThy-3d        TGF--WSPVQCL------GSMEDTTNASNGTNGTAPAIAEPPAPVGV-CWCAD-KKGAPV
TcThy-2f        TGN--WETVQCL------------------------------EHVGV-CWCVT-PQGEPL
CfThy-1e        SGT--WEPVQCL------------------------------EHVGV-CWCVN-RKGQPI
AmThy-2e        NGM--WEPVQCL------------------------------EHVGV-CWCVN-GKGQPM
NvThy-2e        TGA--WEAVQCL------------------------------EHVGV-CWCVN-KKGEPI
NvThy-2c        DGS--YRALQCQ---------------------------------EKNCSCVD-EYGASL
CfThy-1c        DGS--FKSLQCH--------------------------------NSTDCNCVN-HRGVIL
AmThy-2c        DGS--YKPLQCH--------------------------------NGT-CSCVN-DRGVVL
BmThy-2b        DGS--FTSQQCA--------------------------------NGR-CWCVD-SFGTEI
TcThy-2c        DGN--YISTQCH--------------------------------KGE-CWCVD-NFGTEI
DdThy-3b        DGQ-KFAARQCS-------------------------------RNGLVCWCVDPELGTKV
BmThy-2a        NGS--FESRQCS-------------------------------RNGLVCWCVD-TDGNKL
DmThy-3b        GGQ--FSPRQCS-------------------------------RNGLVCWCVDPRTGHKI
AgThy-3b        GGS--FSTRQCS-------------------------------RNGLVCWCVDPKTGNKL
TcThy-2b        DGQ--FEPKQCS-------------------------------RNSLVCWCVD-RMGRKI
CfThy-1b        DGG--FETRQCS-------------------------------RNGLVCWCVD-DEGRKI
NvThy-2b        DGE--YERRQCS-------------------------------RNGLVCWCVD-ALGQKV
AmThy-2b        DGG--YESRQCS-------------------------------RNGLVCWCVD-NNGRKI
DpThy-3d        DGA--FEAVQCD-------------------------------PVTRACWCVS-ADGREL
CeThy-3a        EGF--YKPEQCH--------------------------------DGN-CWCVD-RYGREF
CeThy-4a        EGF--YKPEQCH--------------------------------DGN-CWCVD-RYGREF
BmThy-2c        DGA--YQEVQCR-------------------------------RSDKTCWCVD-TAGNEI
BmThy-2e        DGS--FSPRQCV--------------------------------RGR-CWCVD-AAGERR
TuThy-3a        DGF--YQPIQCH--------------------------------LHK-CWCVD-RYGIEI
HsThy-1a        HGH--YAPTQCH------------------------------GSTGY-CWCVD-RDGREV
TcThy-2d        EGA--YEPKQCN-------------------------------PGTNECWCVD-WRGFEI
NvThy-2d        DGQ--FEPVQCH--------------------------------AGM-CWCVD-EEGREA
CfThy-1d        NGV--FEPVQCH--------------------------------NGV-CWCVD-EEGKEA
AmThy-2d        NGA--FEPVQCH--------------------------------NGM-CWCVD-KKGREA
IsThy-1a        EGY--FMPAQCH------------------------------SSAGM-CWCVD-RHGAEF
BmThy-1a        RGF--YRPRQCH------------------------------AALGV-CWCVD-AHGVEL
DpThy-1a        DGF--FLPTQCH------------------------------TAVGT-CWCVD-KHGVEQ
NvThy-1         RGY--YQSTQCH------------------------------RGLGL-CWCVD-QHGIEF
CfThy-2a        RGY--YRSTQCH------------------------------RGLGL-CWCVD-PHGVEF
AmThy-1a        RGY--YRSTQCH------------------------------RGLGL-CWCVD-PHGVEF
ApThy-1a        EGY--YEHTQCH------------------------------SSVGM-CWCVD-KHGVEV
PhThy-1a        QGY--FRSTQCH------------------------------TSTGM-CWCTD-KHGVEY
DmThy-1a        QGF--YKPTQCH------------------------------NSVGV-CWCVD-KHGVEF
AaThy-1a        QGF--YKPTQCH------------------------------QAVGV-CWCVD-EHGVEF
AgThy-1a        QGF--YKPTQCH------------------------------QAVGV-CWCVD-EHGVEF
CqThy-1a        QGF--YKPTQCH------------------------------QAVGV-CWCVD-EHGVEF
TcThy-1a        QGY--YRPTQCH------------------------------SAIGM-CWCVD-KHGVEF
                 .   :    .                                     . *     *

TuThy-5a        FGTVESV---NGSNMTC----------
TuThy-5d        SNVIS-----SSVI-------------
TuThy-4b        GARVTIP---RDSS-E-----------
TuThy-4c        DYERYNV---TLKD-------------
AgThy-5b        GVERPEK---DKQRLPC----------
AgThy-5b        SDLRN-----VTRGLSA----------
AgThy-4a        FGWDWYRNTERMTC-AC----------
TuThy-5c        ANYRIDRYGNDDRTMKC----------
AgThy-5a        FGEAVHTASIQISM-RC----------
TuThy-5b        SPLVLID---AVSSLSC----------
TuThy-1a        RAPSK-----KIKACDC----------
DmThy-3e        QSTHVFG---AGDR-RC----------
AgThy-3e        PLSSTFK---RGQR-SC----------
DpThy-4a        ANTTQFL---RGEQ-TC----------
```

```
DpThy-5a        ANTTQFL---RGEQ-TC----------
TcThy-2g        LHTNTFK---KGEI-TC----------
NvThy-2f        VGSEPFV---KGTN-IC----------
CfThy-1f        IGSEPFL---KGTS-IC----------
AmThy-2f        IGSEPFL---KGT--NI----------
AgThy-4b        LGTTV-----VHESLWE----------
TuThy-1b        GEKSR-----TLTAEGC----------
DpThy-3f        DDSFT-----RGSV-RC----------
TuThy-2b        PGISSKL---PAKP-DC----------
DmThy-2a        ADDNK-QFRRKGKL-RC----------
TuThy-2a        WKTSA-K--GDKASTDC----------
CeThy-2a        LGSST-T---RGKP-KC----------
ApThy-2a        ANTIV-R---GQKP-KC----------
IsThy-2a        SGSSV-Q---NRRP-NC----------
DpThy-2b        PGTSV-Q---NSKPTNC----------
ApThy-2b        PGTSV-K---DSNP-KC----------
DmThy-2b        PGTSV-K---NKRP-QC----------
NvThy-3b        PGTSS-K---DLTP-NC----------
AmThy-3b        PGTSS-K---NHTP-NC----------
CfThy-3a        PGTSS-K---NRTP-NC----------
TcThy-3b        PGTSV-K---NGKP-KC----------
PhThy-2b        PGTSV-K---DQLP-KC----------
AgThy-2b        PGTST-K---DEKP-VC----------
AaThy-2b        PGTST-K---DKRP-QC----------
IsThy-2b        RGIAT-L---GVKP-DC----------
PhThy-2a        PNSAK-R---HDKP-NC----------
DpThy-2a        PGSSI-R---HNKP-NC----------
AgThy-2a        PNTMV-Q---HGKP-IC----------
AaThy-2a        PNTTV-Q---HGKP-VC----------
TcThy-3a        PYTSV-RWRPDAKP-HCGRKKKSTRRR
RpThy-1a        PNTSV-R---HARP-K-----------
NvThy-3a        PNSTV-R---EGRP-RC----------
CfThy-4a        PDTSV-K---HKKP-RC----------
AmThy-3a        PDTSV-R---NERP-RC----------
TuThy-4a        FGDFL-----DSQ--------------
DpThy-3c        AGTRGVA---SVQQ-RS----------
DmThy-3c        PGTRVKS---PATP-KC----------
AgThy-3c        SGTRT-N---NGQP-NC----------
CeThy-1a        PNSRTRD---GTRP-DC----------
DpThy-3a        AGTRARS---LQLV-NC----------
NvThy-2a        AGTRAPQ---RRLV-DC----------
CfThy-1a        PGTRALN---KNDI-DC----------
AmThy-2a        PGTRENS---IDAI-DC----------
TcThy-2a        PGTRAPA---AALV-NC----------
DmThy-3a        PGSRNET---RTGV-VC----------
AgThy-3a        TGSRRSH---ADDV-NC----------
DmThy-3d        KGTLT-R---ESEP-IC----------
BmThy-2d        KGSLV-R---GSKP-SC----------
DpThy-3e        KGSLT-----RGSP-TC----------
AgThy-3d        KGSLT-K---GSEP-KC----------
TcThy-2f        KGTLT-R---GAQP-LC----------
CfThy-1e        KGSLI-R---GTEP-KC----------
AmThy-2e        KGSLT-R---APEP-KC----------
NvThy-2e        KGSLT-R---DAEP-KC----------
NvThy-2c        KSPVE-----PAST-DC----------
CfThy-1c        KTGVG-----PTASADC----------
AmThy-2c        KSSV------NRSS-DCK---------
BmThy-2b        PETSTHN---ASAV-DC----------
TcThy-2c        PRTR------GTTQ-NC----------
DpThy-3b        KGSMG-S---AQDV-VC----------
BmThy-2a        RGSMG-P---SATV-HC----------
DmThy-3b        KETMG-A---ANNV-NC----------
AgThy-3b        KGTMG-A---AATV-SCEVVENMIGGR
TcThy-2b        RGSMG-P---AGNT-NC----------
CfThy-1b        SGSMG-P---SKKI-DC----------
NvThy-2b        SGSMG-P---AEKV-DC----------
AmThy-2b        SGTMG-P---ADKV-DC----------
DpThy-3d        AGTRVPP---GLQP-QC----------
CeThy-3a        DKSRV-Q---NTLP-DC----------
CeThy-4a        DKSRV-Q---NTLP-DC----------
BmThy-2c        PGTRT-S---NSTP-TC----------
BmThy-2e        HHA-------GPVP-------------
TuThy-3a        ENTRQ-----TEIP-DCD---------
HsThy-1a        EGTRTRP---GMTP-PC----------
TcThy-2d        SKTRT-----NSQL-SC----------
NvThy-2d        AGTRVVE---GLLP-KC----------
CfThy-1d        AGTRVLE---GIVP-RC----------
```

```
AmThy-2d        AGTRVLE---GIVP-KC----------
IsThy-1a        ANTRR-----RDRP-DC----------
BmThy-1a        PGSRT-----KGAP-AC----------
DpThy-1a        NGSRA-----RGKP-DC----------
NvThy-1         AGTRV-R---GTKP-DC----------
CfThy-2a        AGTRT-R---GTRP-DC----------
AmThy-1a        AGTRT-R---GSKP-D-----------
ApThy-1a        PNSRV-----RGKP-NC----------
PhThy-1a        ANTRI-----RGMP-NC----------
DmThy-1a        ANTRT-----RGKP-NC----------
AaThy-1a        ANTRT-----RGKP-NC----------
AgThy-1a        ANTRT-----RGKP-NC----------
CqThy-1a        ANTRT-----RGKP-NC----------
TcThy-1a        ANTRT-----HAKP-NC----------
```

C

```
TuCPI-1         -------MIIVLRNL---------------------------------IFLFSFICLGSS
TuCPI-12        ------------MKL----------------------------------IIFT--CL-FA
TuCPI-7         ------------MKL---------------------------------VNLIIFTCL-FA
TuCPI-6         ------------MKM---------------------------------KLILIV-ILSFG
TuCPI-2         ------------MKV---------------------------------ILALAI-CL-IG
TuCPI-4         ------------MKL---------------------------------ILVIAV-CL-IG
TuCPI-3         ------------MKL---------------------------------ILVIAV-CL-IG
TuCPI-5         ------------MKL---------------------------------ILVIAV-CF-IG
TuCPI-10        MKSDKYKKIVTKMKF----------------------------------FMLTV-CL-FG
TuCPI-13        ------------MKF----------------------------------FMLTV-CL-FG
TuCPI-23        ------------MQF----------------------------------FMLTV-CL-FG
TuCPI-24        ------------MKF----------------------------------FMLTV-CL-FG
TuCPI-14        ------------MKF----------------------------------FILIV-CL-FG
TuCPI-25        ------------MKF----------------------------------FILIV-CL-FG
TuCPI-9         ------------MKF----------------------------------FILTI-CL-FG
TuCPI-16        ------------MKF----------------------------------LILSV-CL-FG
TuCPI-11        ---------MVVSKFINLILERSDLKIDTYFKQLITENEDINACFKNEILHLNI-CL-FA
TuCPI-17        ------------MKF----------------------------------FILTV-CL-FG
TuCPI-8         ------------MKF----------------------------------VVLLI-CL-FS
TuCPI-15        ------------MKL---------------------------------AIILTA-CL-FG
TuCPI-20        ------------MKF----------------------------------LMIAV-CL-FG
TuCPI-19        ------------MKF----------------------------------LMIAV-CL-FG
TuCPI-18        ------------MKF----------------------------------IIIAV-CL-FG
TuCPI-22        ------------MKF----------------------------------ILFAV-CL-IG
TuCPI-21        ------------MKF----------------------------------ILFAV-CL-IG
                             :.                                    :    :  .

TuCPI-1         QSIPKHGHRLV--------------------GGWMPKDIDYEPAKDNAKYAAKLINDQSN
TuCPI-12        AVWTNESGQISETTLIESEDDFRQAPAKNLFGPWVTVPVDDPIIQKYTEQALERQNKEFG
TuCPI-7         AVWTNESGQISETTLIELEDNFRHASTK---GPWVPVPVDDPIIQKYAEEAVETRNKKYD
TuCPI-6         PFLTL----AGI-------------------GEWGRVSMDNPTVLELAELAVDDHNKLST
TuCPI-2         VSEAL----L---------------------GGWRDVDVDNQTVHLLSQMAINHRNSDED
TuCPI-4         ASHQF----ILD-------------------GGWGSVDANSETIKDLAQVATEHRNSQIN
TuCPI-3         ASHQF----RF--------------------GGWQTSDANSETIKDLAQVATEHRNSQIN
TuCPI-5         ASHQF----RF--------------------SGWQTSDANSGTIKDLAQVATEHRNSQIN
TuCPI-10        TVFAK----SLS-------------------TEWTPLPSDDPTVVKFANLAVADINGKEK
TuCPI-13        TVFAK----SPS-------------------TEWKPLPVDDPTVIKLANQAVAHINEKWE
TuCPI-23        TVFAK----SPS-------------------TEWTPLPVDDPTVIKLAKQAVADVNSQEK
TuCPI-24        TVFAK----SPS-------------------TEWTPLPVDDPTVIKLAKQAVANVNSQEK
TuCPI-14        TVFAQ----SHP-------------------VKYKPLPVDDSTVIKLANQAVAKINAEGN
TuCPI-25        TVFAQ----SHP-------------------VKYKPLPVDDSTVIKLANQAVAKINAEGN
TuCPI-9         TVFAE----NHPM------------------PEWKPLPVDDPTVIKLADLAVVDINAKEN
TuCPI-16        ALFAT----SPP-------------------IEWTPLPVDDPTVITLADQAVAYINAQDN
TuCPI-11        TVFAV----TLF-------------------GPWTPLPVDDPTVIKLADQAVVDINAQDN
TuCPI-17        TVFSV----TLL-------------------GPWTPLPVDDPSVIKLAKQAVVDINAHDK
TuCPI-8         VVIARP-------------------------SVWKSIPVDDPTVNLLTEKGIEHRNKNDN
TuCPI-15        TVFAQE---GLP-------------------GGWNSLSTDDSTVNQLAIKSVNHHNSVNN
TuCPI-20        AAWSAVPFEPIE-------------------GAWSSNSVDDPLVAELAAKGLDYENRYGN
TuCPI-19        AAWSAV---TID-------------------GAWSSNSVDDPLVVELAAKGLDYENRYGN
TuCPI-18        AAFAT----SIE-------------PTKHFLGGWKILPVDDPTVVQLAAKGVECYNKNSN
TuCPI-22        AVFAI----EMD-------------------GGWSSISVDHPTVIQLAAKGVEHHNKIAN
TuCPI-21        VVFA------MP-------------------GGWSSLSVDHPTVIQLAAKGVEHHNKIAN
                                                 :     :       :  .    *

TuCPI-1         DMYFQNLIHIHDVKSQV--VGGVKYNITFDMSKTICRKNEIDSDKPEQCVPDRNATIKR-
TuCPI-12        D-RYKRLVSINQAKRQT--LSGYRFEIEMIIRETDCNKNDPKKH---QCQFNSARAPEP-
TuCPI-7         G-HYKRLMSVDVAKKQF--ISGYRFEIEMTIRETECHQNDPKKH---QCQFNSARAPEP-
TuCPI-6         NDYYFKLVKITSVSFQA--LNGIKYSITFIIGQTKCFKTDPNHK---KCDLLHKNINIMN
TuCPI-2         TLYYRKLVNVESARMQV--VSGLKYEVTLVIGETHCAKEDEAAM---LCQVEPNSLREK-
TuCPI-4         SLYYRTLVEIKSAKQQV--VNGMKYELTLVLADTNCAKQDAGAK---LCPVGQGAAKEE-
TuCPI-3         SLYYRTLVEIKSAKQQV--VNGMKYELTLVLADTNCAKQDAGAK---LCPVGQGAAKEE-
TuCPI-5         SLYYRTLVEIKSAKQQV--VNGMKYELTLVLADTNCAKQDAGAK---LCPVGQGAAKEE-
TuCPI-10        L-FYNKLIQIKEAKSS----KAKFYVFKVIVGKTDCPIRGPYTD---ACQIKDDAPRKE-
TuCPI-13        KLFYNKLIEIKEAKSVA--DDGITYMLKMIIRLTYCPKSKPYHD---DCEIRQASSAQI-
TuCPI-23        F-TYNKLIVITEAKSVV--DDGVTYMLKMIIQKTYCPLSKPYHD---DCEINQGLPGST-
TuCPI-24        F-NYNKLIEIQEPKSVV--DDGVTYMLKFIIRSTYCPISKPYHD---DCEI-RELPGRI-
TuCPI-14        GKFYNKLIEIKEARSKL-RHSKITYTIKVILRKTYSHKSKPYTD---ACGINDTAPPKL-
TuCPI-25        GKFYNKLIEIKEARSKL-RHSKITYTIKVILRKTYSHKSKPYTD---ACGINDTAPPKL-
TuCPI-9         SSYYNKLVQIRAAKSRL--MSGIEYELRLDIQKTDCPKSKPYTD---ACQINVTTLPPI-
TuCPI-16        SLYYNKLIQIKEAKSRV--ADKVEYELKLVIRITDCPKSKPYTD---ACQINQDEPPKL-
TuCPI-11        SLYYNKLILIRD-KRRV--GDKIEYELKLVIRQTDCPKSKPYTD---TCQINQDTLPQI-
TuCPI-17        SLYYNKLIEIREAKSRV--ADKIEYELKLVIRITDCPKSKPYTD---ACQINQDLPPKL-
TuCPI-8         SIYYEKLIAIKKAQTQA--LAPAKYKIEFLIGPTECLKTDPNSA---SCQISTNKASET-
TuCPI-15        SAYYKKLVKIQEARYQV--VAGFKYEIKFLIGKTECAKTGNYTD---SCQVAVNSPTEL-
```

```
TuCPI-20        SFYYKKLITIKEARIQAAKPYGVNHEVKLLIGQTDCAKWNVNAT---SCEVSPNAIPEL-
TuCPI-19        SFNYKKLITIKEARAQADKVSGINHEVKLLIGQTDCAKWNANAT---SCEVSPNATPEL-
TuCPI-18        NIYYNKLIKIKKAASEI--VAGMLYEIKFLIGATDCVKSEPDAS---SCKVSPNAIPKL-
TuCPI-22        NLYYKKLITIKEAKSQV--VAGMNYEVKFLIGKTECVKSDANAA---SCEVSANAIPEL-
TuCPI-21        NLYYKRLISIEEAKSQV--VAGINYEVKFLIGKTECVKSDANAA---SCEVSANAIPEL-
                   :  *: :              . . . :  * .            *

TuCPI-1         -CYAVVYERPWE-SKRQLLDHKCNNHLSYSEYFNDTNIAREQEYQQNRKMNLLIEHFT--
TuCPI-12        -VVFDVWVKGD-------------------------------------------------
TuCPI-7         -VVFDVWVNTRN-Q----------------------------------------------
TuCPI-6         LCSYLFWIKPGTPKTVEIIHHACDKGHQLRMQDYVGHEYSVEGYEAHEDTEDGQDDDYVA
TuCPI-2         -CVYTFWLEAKT-QNTNIVTSSCSQL----------------------------------
TuCPI-4         -CVYTIWIESTK-ETPEVTSSSCTDL----------------------------------
TuCPI-3         -CVYTIWVESTK-EEPAVTSSSCTDL----------------------------------
TuCPI-5         -CVYTIWIESTK-EAPEVTSSSCTDL----------------------------------
TuCPI-10        -CSVYYYYNT---VEWKHGTYTCDELKSS-------------------------------
TuCPI-13        -CTVEGHKPSGS-EEIKISNLSCDTIPLPPS-----------------------------
TuCPI-23        -CIVDAHKPVGS-EEIKIGRLQCDEIPQPPS-----------------------------
TuCPI-24        -CSVDAHKPVGS-EEIKIGRLQCDEIPLPPS-----------------------------
TuCPI-14        -CTIDAYVRVGS-DESKIVILRCETIHKPVK-----------------------------
TuCPI-25        -CTIDAYVRVGS-DESKIVILRCETIHKLVK-----------------------------
TuCPI-9         -CTVHLHEDPGS-KEIKITAFQCDSAQDHE------------------------------
TuCPI-16        -CTIDAYVRAGS-EENKIYKFLCGEI----------------------------------
TuCPI-11        -CTYELFSRAG--SKNKITTLQCDELKSYMIWKKRNLRFKISRFQKKIRERFA-------
TuCPI-17        -CTYELFVRAG--SKNKFTTLQCDELKSA-------------------------------
TuCPI-8         -CIFVFLIRRGS-NDIHITRDFCSPA----------------------------------
TuCPI-15        -CTYVFWMPPV--DKDRITSFDCVATNEDVLEKHLTINCEVFIN----------------
TuCPI-20        -CTYVTWVSPDL-HWRELTQASCESAE---------------------------------
TuCPI-19        -CTYVIWVSPDL-HWRSLMQASCESAE---------------------------------
TuCPI-18        -CTYHFWIKSWS-GFEQITQVSCVPV----------------------------------
TuCPI-22        -CTYVFWVRPGS-DNAQITQASCAPAK---------------------------------
TuCPI-21        -CTYVFWVQPGS-DNAQITQASCGPAK---------------------------------


TuCPI-1         ----
TuCPI-12        ----
TuCPI-7         ----
TuCPI-6         YDND
TuCPI-2         ----
TuCPI-4         ----
TuCPI-3         ----
TuCPI-5         ----
TuCPI-10        ----
TuCPI-13        ----
TuCPI-23        ----
TuCPI-24        ----
TuCPI-14        ----
TuCPI-25        ----
TuCPI-9         ----
TuCPI-16        ----
TuCPI-11        ----
TuCPI-17        ----
TuCPI-8         ----
TuCPI-15        ----
TuCPI-20        ----
TuCPI-19        ----
TuCPI-18        ----
TuCPI-22        ----
TuCPI-21        ----
```
