# Supplementary figures and images for: Cysteine peptidases and their inhibitors in Tetranychus urticae: a comparative genomic approach
Source: BMC Genomics. 2012 Jul 11;13:307. doi: 10.1186/1471-2164-13-307 (PMC3407033; doi:10.1186/1471-2164-13-307)

B

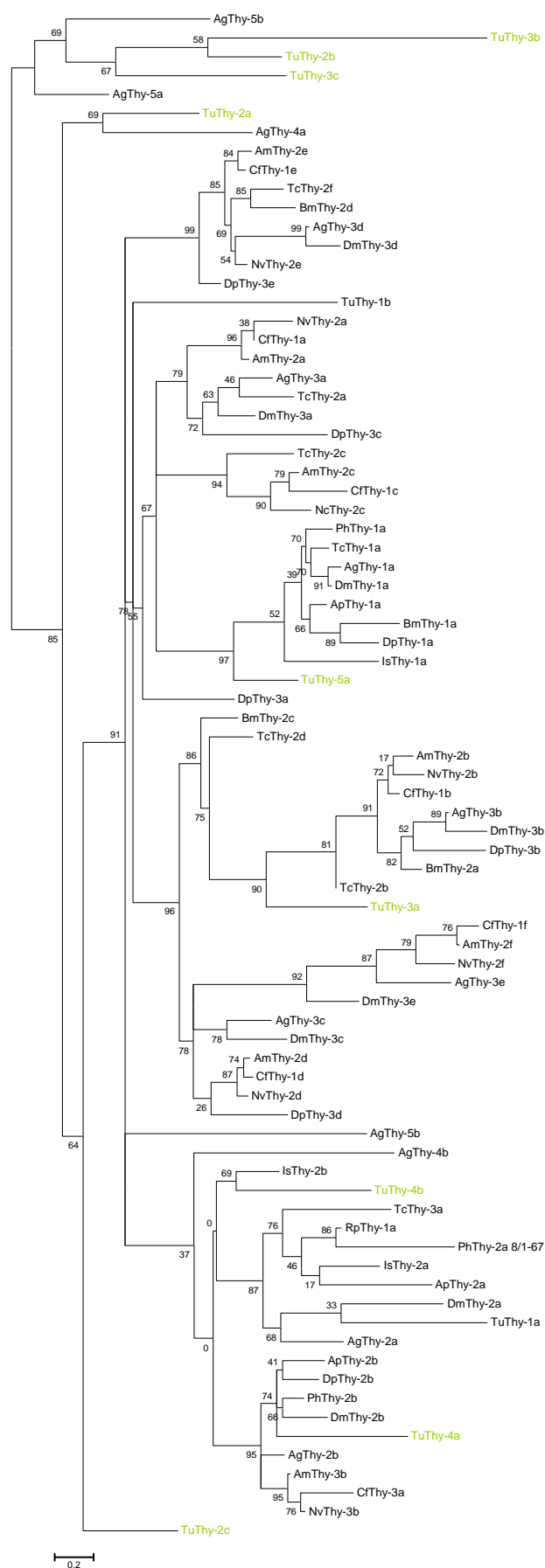

Supplement: Additional file 2 — Complete phylogenetic trees of the cystatin (A) and thyropins (B) domains from selected arthropod species. [file 1471-2164-13-307-S2.pdf]
