# Supplementary material for: Cysteine peptidases and their inhibitors in Tetranychus urticae: a comparative genomic approach
Source: BMC Genomics. 2012 Jul 11;13:307. doi: 10.1186/1471-2164-13-307 (PMC3407033; doi:10.1186/1471-2164-13-307)

Additional file 3. Domain architectures of the proteins with thyropin domains from the selected arthropod species. Ag, *Anopheles gambiae*; Am, *Apis mellifera*; Ap, *Acyrtosiphon pisum*; Bm, *Bombyx mori*; Cf, *Camponotus floridanus*; Dm, *Drosophila melanogaster*; Dp, *Daphnia pulex*; Is, *Ixodes scpularis*; Nv, *Nasonia vitripennis*; Ph, *Pediculus humanus*; Rp, *Rhodnius prolixus*; Tc, *Tribolium castaneum*; Tu, *Tetranychus urticae*. Thy, thyropin; Ant, antistasin; WAP, whey acidic protein; SPARC, secreted protein acidic and rich in cysteine.

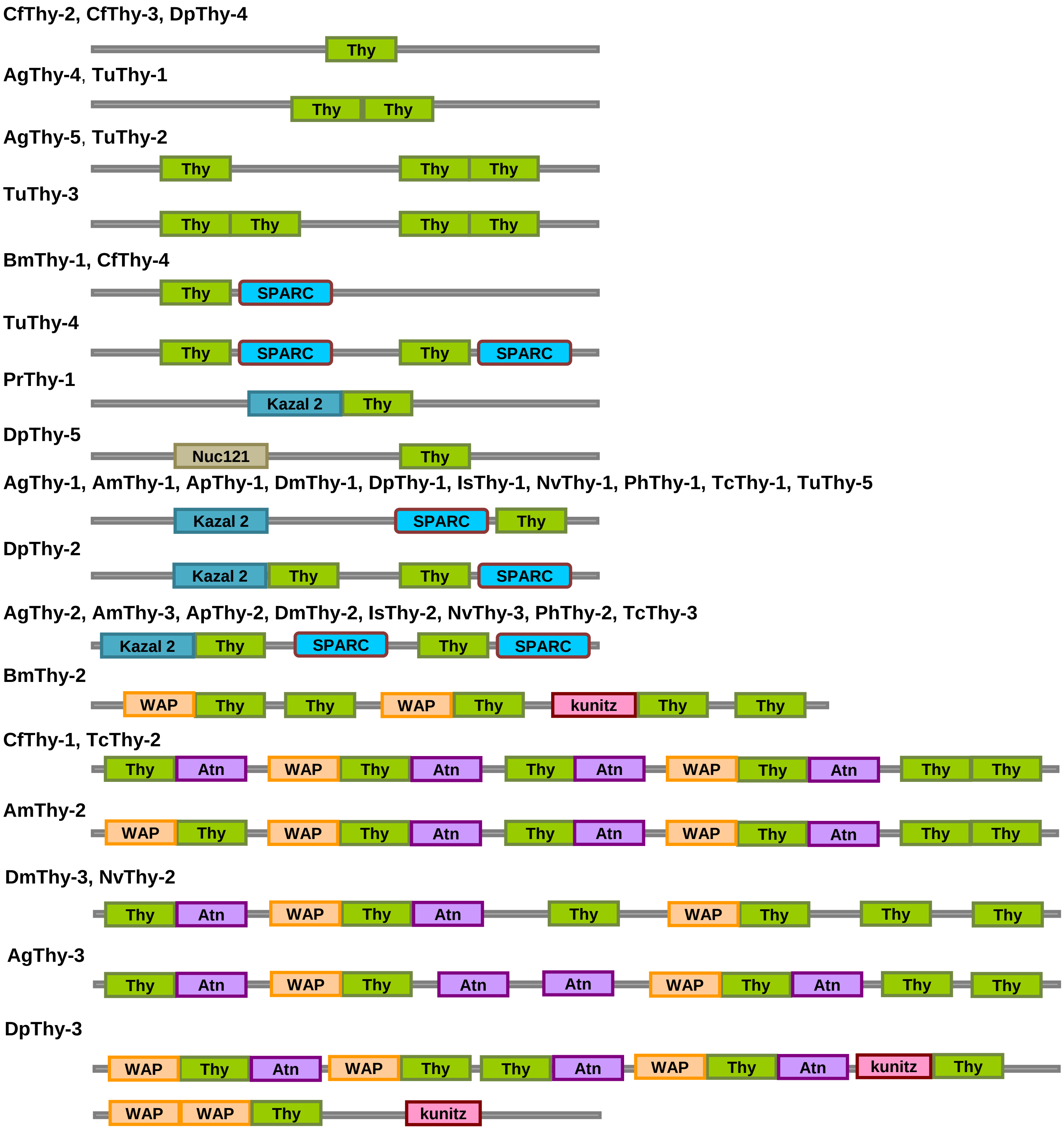

Supplement: Additional file 3 — Domain architectures of the proteins with thyropin domains from the selected arthropod species. [file 1471-2164-13-307-S3.pdf]
