# Supplementary material for: Cysteine peptidases and their inhibitors in Tetranychus urticae: a comparative genomic approach
Source: BMC Genomics. 2012 Jul 11;13:307. doi: 10.1186/1471-2164-13-307 (PMC3407033; doi:10.1186/1471-2164-13-307)
Supplement: Additional file 4 — Alignment of the different four groups of cystatin sequences from T. urticae showing conserved motifs and structural features. [file 1471-2164-13-307-S4.ppt]

## Slide 1
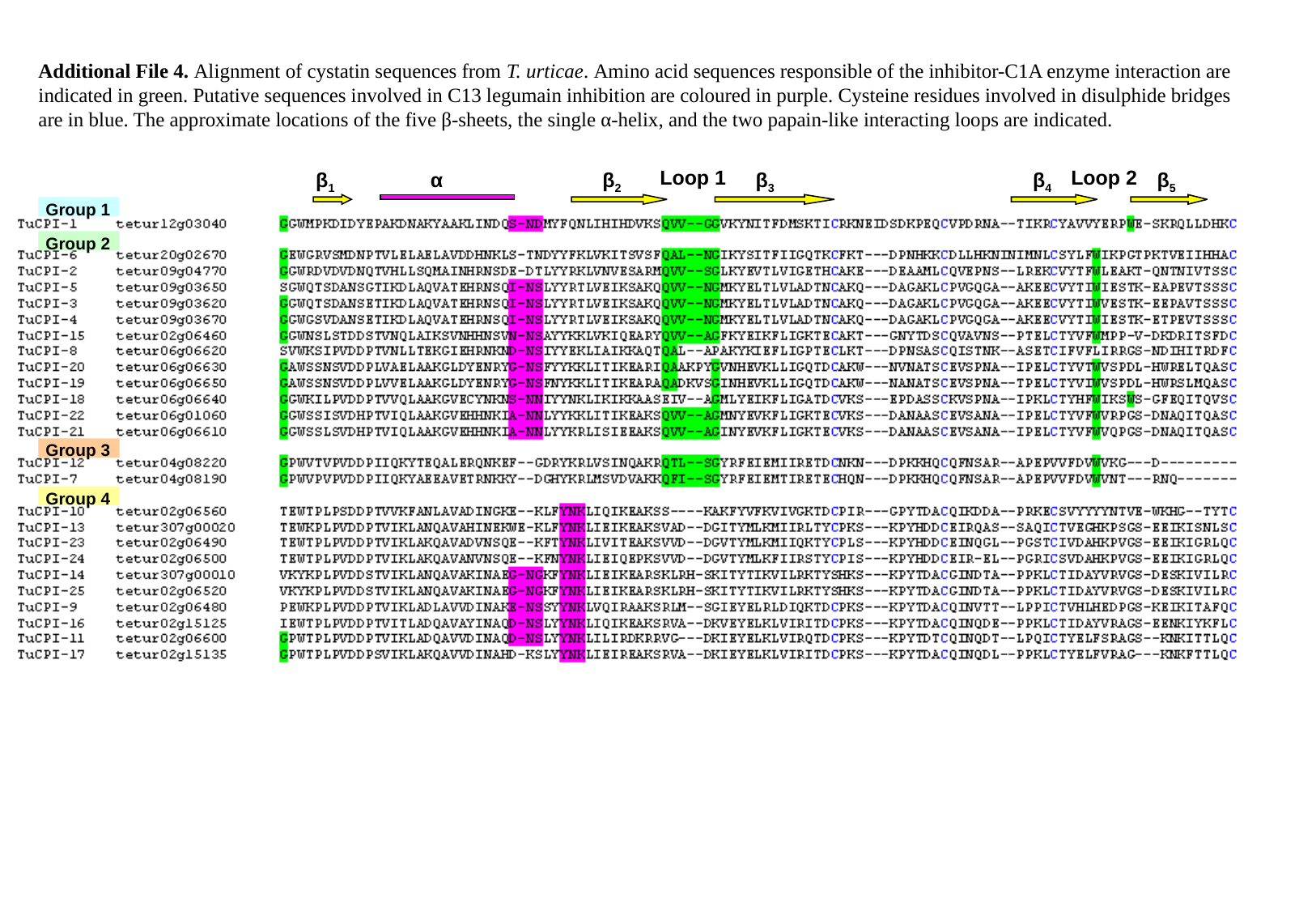

Additional File 4. Alignment of cystatin sequences from T. urticae. Amino acid sequences responsible of the inhibitor-C1A enzyme interaction are indicated in green. Putative sequences involved in C13 legumain inhibition are coloured in purple. Cysteine residues involved in disulphide bridges are in blue. The approximate locations of the five β-sheets, the single α-helix, and the two papain-like interacting loops are indicated.
Loop 1
Loop 2
β1
α
β2
β3
β4
β5
Group 1
Group 2
Group 3
Group 4
