# Supplementary material for: Cysteine peptidases and their inhibitors in Tetranychus urticae: a comparative genomic approach
Source: BMC Genomics. 2012 Jul 11;13:307. doi: 10.1186/1471-2164-13-307 (PMC3407033; doi:10.1186/1471-2164-13-307)
Supplement: Additional file 5 — Information about gene models and accession numbers corresponding to the proteins (cystatins, thyropins, C1A peptidases and C13 peptidases) used in this study. [file 1471-2164-13-307-S5.pdf]

Additional file 5. addfile5.doc

Information about gene models and accession numbers corresponding to cystatins (CPI), stefins (STF) and thyropins (Thy) used in this study. Ag, *Anopheles gambiae*; Am, *Apis mellifera*; Ap, *Acyrtosiphon pisum*; Bm, *Bombyx mori*; Cf, *Camponotus floridanus*; Dm, *Drosophila melanogaster*; Dp, *Daphnia pulex*; Is, *Ixodes scpularis*; Nv, *Nasonia vitripennis*; Ph, *Pediculus humanus*; Rp, *Rhodnius prolixus*; Tc, *Tribolium castaneum*; Tu, *Tetranychus urticae*.

**Protein**	**Organism**	**Gene model/Accession number**
AgCPI-1	*Anopheles gambiae*	AGAP002878
AmCPI-1	*Apis mellifera*	GB13004
ApCPI-1	*Acyrthosiphon pisum*	ACYPI000376
BmCPI-1	*Bombyx mori*	BGIBMGA005131
CfCPI-1	*Camponotus floridanus*	08100--XP_392381.2
DmCPI-1	*Drosophila melanogaster*	CG12163
DmCPI-2	*Drosophila melanogaster*	CG31313
DmCPI-3	*Drosophila melanogaster*	CG8050
DmCPI-4	*Drosophila melanogaster*	CG8066
DmCPI-5	*Drosophila melanogaster*	CG15369
DpCPI-1	*Daphnia pulex*	hxAUG26res89g7t1
DpCPI-2	*Daphnia pulex*	hxAUG26rep2s2g239t1
DpCPI-3	*Daphnia pulex*	hxNCBI_GNO_980014
DpCPI-4	*Daphnia pulex*	hxAUG26us79g105t1
DpCPI-5	*Daphnia pulex*	hxAUG26up2s1g144t1
DpCPI-6	*Daphnia pulex*	hxAUG26us113g59t1
DpCPI-7	*Daphnia pulex*	hxAUG26rep2s1g141t1
DpCPI-8	*Daphnia pulex*	hxAUG26up2s1g147t1
DpCPI-9	*Daphnia pulex*	hxAUG26res74g50t1
DpCPI-10	*Daphnia pulex*	hxAUG25p2s1g223t1
IsCPI-1	*Ixodes scapularis*	ISCW018603
IsCPI-2	*Ixodes scapularis*	ISCW018602
IsCPI-3	*Ixodes scapularis*	ISCW018601
IsCPI-4	*Ixodes scapularis*	ISCW018604
IsCPI-5	*Ixodes scapularis*	ISCW011771
IsCPI-6	*Ixodes scapularis*	ISCW002216
IsCPI-7	*Ixodes scapularis*	ISCW002036
IsCPI-8	*Ixodes scapularis*	ISCW002037
IsCPI-9	*Ixodes scapularis*	ISCW017861
IsCPI-10	*Ixodes scapularis*	ISCW024528
IsCPI-11	*Ixodes scapularis*	ISCW010785
NvCPI-1	*Nasonia vitripennis*	NV12850-PA
PhCPI-1	*Pediculus humanus*	PHUM113300
PhCPI-2	*Pediculus humanus*	PHUM345820
RpCPI-1	*Rhodnius prolixus*	RPRC012888
RpCPI-2	*Rhodnius prolixus*	RPRC012888
RpCPI-3	*Rhodnius prolixus*	RPRC012888
RpCPI-4	*Rhodnius prolixus*	RPRC012888
TcCPI-1	*Tribolium castaneum*	TC009844
TuCPI-1	*Tetranychus urticae*	tetur12g03040
TuCPI-2	*Tetranychus urticae*	tetur09g04770
TuCPI-3	*Tetranychus urticae*	tetur09g03620

TuCPI-4	*Tetranychus urticae*	tetur09g03670
TuCPI-5	*Tetranychus urticae*	tetur09g03650
TuCPI-6	*Tetranychus urticae*	tetur20g02670
TuCPI-7	*Tetranychus urticae*	tetur04g08190
TuCPI-8	*Tetranychus urticae*	tetur06g06620
TuCPI-9	*Tetranychus urticae*	tetur02g06480
TuCPI-10	*Tetranychus urticae*	tetur02g06560
TuCPI-11	*Tetranychus urticae*	tetur02g06600
TuCPI-12	*Tetranychus urticae*	tetur04g08220
TuCPI-13	*Tetranychus urticae*	tetur307g00020
TuCPI-14	*Tetranychus urticae*	tetur307g00010
TuCPI-15	*Tetranychus urticae*	tetur02g06460
TuCPI-16	*Tetranychus urticae*	tetur02g15125
TuCPI-17	*Tetranychus urticae*	tetur02g15135
TuCPI-18	*Tetranychus urticae*	tetur06g06640
TuCPI-19	*Tetranychus urticae*	tetur06g06650
TuCPI-20	*Tetranychus urticae*	tetur06g06630
TuCPI-21	*Tetranychus urticae*	tetur06g06610
TuCPI-22	*Tetranychus urticae*	tetur06g01060
TuCPI-23	*Tetranychus urticae*	tetur02g06490
TuCPI-24	*Tetranychus urticae*	tetur02g06500
TuCPI-25	*Tetranychus urticae*	tetur02g06520
DpSTF-1	*Daphnia pulex*	hxAUG25p2s3g32t1
TuSTF-1	*Tetranychus urticae*	tetur32g02347

**Protein**	**Organism**	**Gene model/Accession number**
AgThy-1	*Anopheles gambiae*	AGAP003400
AgThy-2	*Anopheles gambiae*	AGAP007489
AgThy-3	*Anopheles gambiae*	AGAP004333
AgThy-4	*Anopheles gambiae*	AGAP005941
AgThy-5	*Anopheles gambiae*	AGAP007053
AmThy-1	*Apis mellifera*	GB11431
AmThy-2	*Apis mellifera*	GB10002
AmThy-3	*Apis mellifera*	GB10197
ApThy-1	*Acyrthosiphon pisum*	ACYPI005271
ApThy-2	*Acyrthosiphon pisum*	ACYPI000745
BmThy-1	*Bombyx mori*	BGIBMGA007989
BmThy-2	*Bombyx mori*	BGIBMGA005119
CfThy-1	*Camponotus floridanus*	05332--XP_001120452.1
CfThy-2	*Camponotus floridanus*	08415--XP_393267.2
CfThy-3	*Camponotus floridanus*	12541--XP_394975.3
CfThy-4	*Camponotus floridanus*	12542--XP_394975.3
DmThy-1	*Drosophila melanogaster*	CG13830
DmThy-2	*Drosophila melanogaster*	CG2264
DmThy-3	*Drosophila melanogaster*	CG5639
DpThy-1	*Daphnia pulex*	hxAUG26up1s10g323t1
DpThy-2	*Daphnia pulex*	hxAUG26rep1s12g108t1
DpThy-3	*Daphnia pulex*	hxAUG26us1668g136t1
DpThy-4	*Daphnia pulex*	hxAUG25s98g63t1
DpThy-5	*Daphnia pulex*	hxAUG25s98g69t1
IsThy-1	*Ixodes scapularis*	ISCW022173
IsThy-2	*Ixodes scapularis*	ISCW019551
NvThy-1	*Nasonia vitripennis*	NV16306
NvThy-2	*Nasonia vitripennis*	NV11533
NvThy-3	*Nasonia vitripennis*	NV12111
PhThy-1	*Pediculus humanus*	PHUM285300
PhThy-2	*Pediculus humanus*	PHUM607640

RpThy-1	*Rhodnius prolixus*	RPRC015636
TcThy-1	*Tribolium castaneum*	TC004567
TcThy-2	*Tribolium castaneum*	TC008734
TcThy-3	*Tribolium castaneum*	TC010525
TuThy-1	*Tetranychus urticae*	tetur09g04580
TuThy-2	*Tetranychus urticae*	tetur08g07040
TuThy-3	*Tetranychus urticae*	tetur40g00090
TuThy-4	*Tetranychus urticae*	tetur32g01640
TuThy-5	*Tetranychus urticae*	tetur03g05950
